# Combining Nanopore direct RNA sequencing with genetics and mass spectrometry for analysis of T-loop base modifications across 42 yeast tRNA isoacceptors

**DOI:** 10.1093/nar/gkae796

**Published:** 2024-09-28

**Authors:** Ethan A Shaw, Niki K Thomas, Joshua D Jones, Robin L Abu-Shumays, Abigail L Vaaler, Mark Akeson, Kristin S Koutmou, Miten Jain, David M Garcia

**Affiliations:** Institute of Molecular Biology, University of Oregon, Eugene, OR 97403, USA; Department of Biology, University of Oregon, Eugene, OR 97403, USA; Department of Bioengineering, Northeastern University, Boston, MA 02115, USA; Department of Chemistry, University of Michigan, Ann Arbor, MI 48109, USA; Biomolecular Engineering Department, University of California Santa Cruz, Santa Cruz, CA 95064, USA; Center for Molecular Biology of RNA, University of California Santa Cruz, Santa Cruz, CA 95064, USA; Institute of Molecular Biology, University of Oregon, Eugene, OR 97403, USA; Biomolecular Engineering Department, University of California Santa Cruz, Santa Cruz, CA 95064, USA; Center for Molecular Biology of RNA, University of California Santa Cruz, Santa Cruz, CA 95064, USA; Department of Chemistry, University of Michigan, Ann Arbor, MI 48109, USA; Department of Bioengineering, Northeastern University, Boston, MA 02115, USA; Department of Physics, Northeastern University, Boston, MA 02115, USA; Khoury College of Computer Sciences, Northeastern University, Boston, MA 02115, USA; Institute of Molecular Biology, University of Oregon, Eugene, OR 97403, USA; Department of Biology, University of Oregon, Eugene, OR 97403, USA

## Abstract

Transfer RNAs (tRNAs) contain dozens of chemical modifications. These modifications are critical for maintaining tRNA tertiary structure and optimizing protein synthesis. Here we advance the use of Nanopore direct RNA-sequencing (DRS) to investigate the synergy between modifications that are known to stabilize tRNA structure. We sequenced the 42 cytosolic tRNA isoacceptors from wild-type yeast and five tRNA-modifying enzyme knockout mutants. These data permitted comprehensive analysis of three neighboring and conserved modifications in T-loops: 5-methyluridine (m^5^U_54_), pseudouridine (Ψ_55_), and 1-methyladenosine (m^1^A_58_). Our results were validated using direct measurements of chemical modifications by mass spectrometry. We observed concerted T-loop modification circuits—the potent influence of Ψ_55_ for subsequent m^1^A_58_ modification on more tRNA isoacceptors than previously observed. Growing cells under nutrient depleted conditions also revealed a novel condition-specific increase in m^1^A_58_ modification on some tRNAs. A global and isoacceptor-specific classification strategy was developed to predict the status of T-loop modifications from a user-input tRNA DRS dataset, applicable to other conditions and tRNAs in other organisms. These advancements demonstrate how orthogonal technologies combined with genetics enable precise detection of modification landscapes of individual, full-length tRNAs, at transcriptome-scale.

## Graphical Abstract

**Graphical Abstract:**
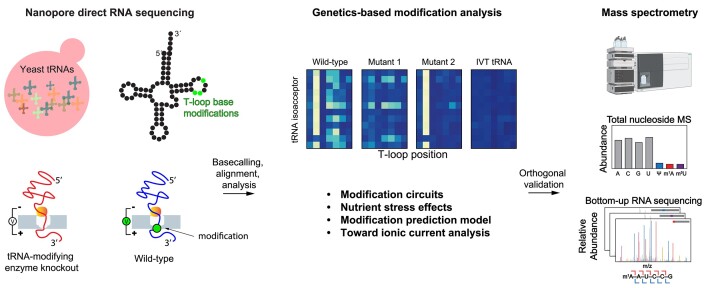

## Introduction

tRNA is the most densely modified RNA species in nature. Biochemical and biophysical methods have long been used to determine tRNA sequences and their modifications per isoacceptor (1,2). Due to cost and time, however, these approaches are not scalable to the transcriptome. More recently, high-throughput sequencing methods that begin with reverse transcription of RNA, followed by PCR, have achieved tRNA sequencing at the transcriptome scale. With few exceptions (3,4), these methods detect modifications individually in each sequenced tRNA. Nanopore direct-RNA sequencing (DRS) permits simultaneous detection of chemically modified nucleosides on individual, full-length, tRNA molecules (5,6).

Previously, we applied DRS to directly sequence full-length *Escherichia coli* tRNA molecules, including all 43 isoacceptors (5). Evidence for nucleotide modifications was observed in sequence miscalls when comparing molecules from wild-type cells with synthetic tRNA controls containing identical canonical (unmodified) nucleotides. We and others have also demonstrated that base miscalls in DRS data can be associated with the presence of specific chemical modifications in mRNA, rRNA and tRNA (5–12). Given the importance of advancing Nanopore-based measurements for RNA (13), particularly for eukaryotic tRNAs, which are even more densely modified than tRNAs in prokaryotes (14), cross-validation with appropriate biological controls is essential.

**Figure 1.**
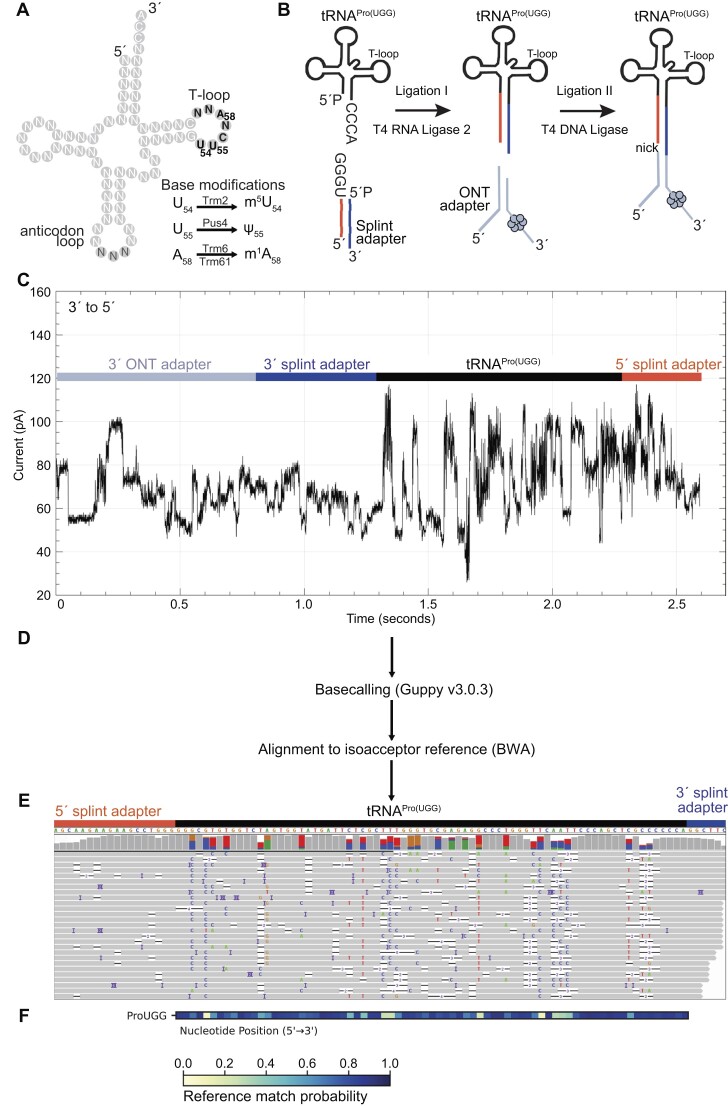
Overview of tRNA library preparation, sequencing, and alignment strategy. (**A**) Illustration of a generic yeast tRNA, highlighting overall structural segments, three chemical modifications that can occur in the T-loop, and the names of the enzymes that catalyze them. (**B**) tRNAs are ligated to a double-stranded splint adapter with RNA Ligase 2 by the tRNA’s 3′ NCCA overhang. A second ligation is performed using T4 DNA Ligase with the tRNA and ONT sequencing adapters. (**C**) Ionic current trace measured in picoamperes for an adapted tRNA^Pro(UGG)^ molecule as it translocates from the 3′ to 5′ direction through a Nanopore. (**D**) Ionic current is basecalled using Guppy v3.0.3. Fastq files are aligned to our *S. cerevisiae* reference sequences using BWA-MEM. (**E**) Alignments are visualized in Integrative Genomics Viewer (32). The reference sequence for tRNA^Pro(UGG)^ is on the top. Read coverage is designated by the height of the gray bar at that position. In the panel containing gray or colored vertical bars, gray represents a match to the reference base for at least 80% of the reads, and colored represents the relative proportion of alternative nucleotide calls at that position. Colored bars (U/T = red; A = green; C = blue, G = gold) indicate positions where base call differs from the listed reference base. Rows below show alignments of individual reads with the reference, interrupted by: non-reference basecalls (colored letters); deletions (white spaces bisected with a black bar); and insertions (purple spaces bisected with a black bar). (**F**) Reference match probabilities are calculated with marginCaller (27) and used to generate a heatmap, where the nucleotide position is shown from 5′–3′ along the tRNA. Dark blue indicates that the base-called nucleotide matches the reference nucleotide more frequently given the alignment, and yellow indicates that the base-called nucleotide matches the reference nucleotide less frequently.

Among >50 distinct chemical modifications in eukaryotic tRNAs (14,15), 25 of them exist in the tRNAs of the budding yeast *Saccharomyces cerevisiae* (14,16). We examined closely-spaced, conserved modifications in the T-loop of 42 cytosolic tRNA isoacceptors in *S. cerevisiae* using Nanopore DRS. We focused on the T-loop (Figure 1A) for three reasons. (i) The T-loop in yeast tRNA isoacceptors contains three conserved chemical modifications: 5-methyluridine (m^5^U, also referred to as ‘T’ or ‘rT’, or ribo-thymidine), pseudouridine (Ψ), and 1-methyladenosine (m^1^A). (ii) These modifications occur in stereotyped positions on tRNAs across kingdoms (17). (iii) T-loop modifications contribute to variations in thermostability for different isoacceptors (18). In budding yeast tRNAs, m^5^U and m^1^A modifications have been documented only in the T-loop at stereotyped positions 54 and 58, respectively (14). In contrast, Ψ has been documented both in the T-loop (position 55) and in other positions in many tRNA isoacceptors (14).

The work herein leverages yeast genetics along with sequence miscall-based machine learning classifiers to predict modifications in the T-loop across 42 yeast isoacceptors. We sequenced tRNA from yeast strains in which each one of the three enzymes responsible for modifying the T-loop was knocked out, as well as an *in vitro* transcribed (IVT) library of the same 42 isoacceptor sequences lacking all modifications. Using these data, we inferred the presence of Ψ_55_ and m^1^A_58_ across all 42 cytosolic tRNA isoacceptors. Moreover, we observed that Ψ_55_ strongly influenced addition of m^1^A_58_ on more isoacceptors than has been previously documented for this modification ‘circuit’ (6,19). A combination of total nucleoside LC–MS/MS analysis and tRNA sequencing by LC–MS/MS as orthogonal methods were used to validate or refute DRS-based inferences of modification status. We also showed that lengthening the adaptors used in library preparation can facilitate ionic current analysis of tRNA. Synthesis of these approaches establishes a rigorous framework for analyzing multiple chemical modifications simultaneously in tRNA, advancing understanding of tRNA structure and function more broadly.

## Materials and methods

### Yeast strains

See Supplementary Table S1 for the list of strains used in this study and their genotypes.

YDG973 was constructed from crossing haploids YDG221 and YDG963 (Supplementary Table S1). A colony from each haploid was patched onto either side of a YPD plate and mixed in the middle in an additional patch. The plate was incubated overnight at 30°C and replica-printed using sterile velvets to SD-Lys, SD-Met and SD-Lys-Met dropout plates to select for diploids. A small globule of the cell mixture from the SD-Lys-Met plate was streaked to single colonies on a new SD-Lys-Met plate to obtain a clonal diploid.

The diploid was sporulated by inoculating a single colony into 3 ml of YPD media and incubating on a roller drum wheel at 30°C for 2 days. The yeast were washed 2× with 1 ml of SPO media before being resuspended in 3 ml of SPO media. The diploids were incubated on a roller drum wheel at 25°C for 6 days. The tubes were collected and 500 μl of sample was added to a microcentrifuge tube and spun down. The supernatant was removed and 200 μl of digestion cocktail (10 μl β-mercaptoethanol, 10 μl zymolyase, 200 μl KPO_4_ solution) was used to resuspend the pellet. The yeast were digested for 8 min at room temperature and then placed on ice after adding an additional 200 μl KPO_4_ to stop digestion. Yeast were spread onto a YPD plate and dissected with the Singer MSM 200 System. The spores were genotyped by PCR to obtain a haploid that was both *pus4*Δ and *trm2*Δ.

For making the Pus4 catalytic mutant and HA-tagged Pus4, CRISPR plasmids targeted to cut at the R286 codon of *PUS4* and at the C-terminus of *PUS4* were made from pJH2972 (http://www.addgene.org/100956). CRISPR plasmid construction followed the protocol as described in (20). The oligos and gBlocks used for construction of the CRISPR plasmids and the repair templates were purchased from IDT. Sequences are shown in Supplementary Table S2.

To create a Pus4-R286K mutant in which the AGG arginine codon at the 286th amino acid position of *PUS4* was replaced with an AAG lysine codon, PDG266 was transformed by lithium-acetate transformation into wild-type BY4741 yeast (YDG1) along with an 83 bp template containing the desired mutation with homology to the surrounding region. The transformation was plated onto an SD-Ura agar plate. The plasmid was counterselected by growth on 5-FOA. Transformants were screened for the presence of the mutation by PCR of the genomic locus followed by Sanger sequencing.

A 3xHA tag was inserted at the C-terminus of *PUS4* using a similar procedure in which PDG342 was transformed into yeast along with a 170 bp template containing the 90 bp sequence of the 3xHA tag with 40bp of homology to the target site on either side. YDG1033 which contains both the R286K mutation and a C-terminal HA tag on *PUS4* was created by using CRISPR to create the R286K mutation in the HA-tagged YDG989. Transformants were screened for the presence of the tag by PCR of the genomic locus followed by Sanger sequencing.

### Yeast growth conditions

Yeast strains were streaked out from the −80°C freezer onto YPD plates and grown for 2 days at 30°C. Three colonies were used to inoculate three culture tubes containing 3 ml of YPD media. The tubes were grown overnight on a roller drum wheel at 30°C. The next morning the cultures were diluted in 5 ml YPD media to an OD_600_ of 0.1. The cultures were grown for ∼6 h at 30°C on a roller drum wheel to an OD_600_ of ∼0.8. The yeast were centrifuged in 15 ml conical tubes at 3000g and 4°C for 2 min. The supernatant was removed, and the yeast were resuspended in 1 ml of cold 1× PBS and transferred to microcentrifuge tubes. The tubes were then centrifuged at 9500 rpm for 1 min at 4°C. The supernatant was poured out, and the microcentrifuge tubes were immediately placed into liquid nitrogen to flash freeze. They were stored at −80°C. To grow yeast to saturation the same protocol was used, except strains were grown for ∼72 h.

### tRNA purification

The yeast pellets, generated from cultures described above, were removed from the −80°C freezer and thawed on ice. The pellets were resuspended with 400 μl of TES buffer made with a final concentration of 10 mM Tris pH 7.5, 10 mM EDTA and 0.5% SDS. To lyse the yeast cells, 400 μl of acidic phenol was added, and the tubes were incubated for 1 h at 65°C with 10 s of vortexing every 15 min. The lysates were placed on ice for 5 min and centrifuged at 13 000 rpm for 10 min at 4°C.The top aqueous layer was transferred to a fresh tube followed by the addition of 400 μl of chloroform. The solution was vortexed for 10 s and centrifuged once again at 13 000 rpm for 10 min at 4°C. These steps were repeated one more time—the aqueous layer was removed to a new tube, chloroform was added, and the tubes were vortexed and centrifuged. The resulting aqueous layer was removed one last time into a clean microcentrifuge tube and 1/10 volume of 3 M NaAc pH5.5, and 2.5 volumes of cold 100% EtOH were added. The tubes were incubated at −80°C for 1 h and centrifuged at 13 000 rpm for 10 min at 4°C. The supernatant was removed and 500 μl of cold 70% EtOH was added. The tubes were centrifuged again at 13 000 rpm for 10 min at 4°C, and the pellet was resuspended in 25 μl of water.

An 8% acrylamide gel was made using National Diagnostics SequaGel 19:1 Denaturing Gel System. The gel was pre-run at 45 mA for 30 min. Total RNA was diluted to 10 μl at 10 μg/μl and 10 μl of 2× RNA Loading Dye (NEB) was mixed with the RNA. The RNA was incubated at 70°C for 8 min and loaded into the gel. The gel was run at 60 mA for 1 h in 1× TBE pH 8.3. It was removed from the gel apparatus and stained with SYBR Gold (Invitrogen) for visualization on an Amersham Typhoon (Cytiva). Using UV shadowing, tRNA was isolated by cutting the gel to select RNA ∼60–85 nt in length. The gel fragments were placed into a microcentrifuge tube with 450 μl of 0.3 M NaCl and incubated on a tube inverter overnight at 4°C. The next day the liquid was transferred to a fresh microcentrifuge tube and 1.05 ml of 100% EtOH was added. The tubes were incubated at −80°C for an hour and then centrifuged at 13 000 rpm for 30 min at 4°C. The supernatant was removed, and the pellet was allowed to air dry for 10 min. The pellet was resuspended in 20 μl of water, and the tRNA was stored at −80°C.

### Nanopore data generation

#### Splint adapter preparation

The RNA and DNA oligonucleotides for the splint adapters were ordered from IDT. Their sequences are shown in Supplementary Table S2. Separate 10μM stock solutions of the five double-stranded splint adapters were assembled in 1× TNE (10 mM Tris, 50 mM NaCl, 1 mM EDTA) by combining equimolar concentrations of the common adapter that abuts the 3′ end of the tRNA, and one of five adapter strands complementary to the 3′ tRNA overhang of ACCA, GCCA, UCCA, CCCA or CCA (see Supplementary Table S2 for sequences). The splints were annealed by heating to 75°C for 1 min and then slow cooling to room temperature.

#### tRNA sequencing

250 ng (∼10 pmol) of total tRNA was ligated to five different double-stranded splint adapters specific to ACCA (8 pmol), GCCA (8pmol), UCCA (8 pmol), CCCA (4 pmol) and CCA (4 pmol) tRNA 3′ overhangs (see Supplementary Table S2 for oligonucleotide sequences). Prior to the ligation reaction, the tRNA was heated to 95°C for 2 min, placed at room temperature for 2 min, then placed on ice for 2 min. The first ligation was done in a total volume of 20 μl and included, in addition to the tRNA and splint adapters, 1× RNA Ligase 2 buffer (NEB), 5% PEG 8000, 6.25 mM DTT, 6.25 mM MgCl2, 2 mM ATP and 0.5 unit/μl T4 RNA Ligase 2 (NEB, stock concentration 10 000 U/ml, a total of 10 units per reaction). The ligation reaction was performed at room temperature for 45 min. The reaction was purified using 1.8× volume of magnetic beads (RNA Clean XP, Beckman Coulter). The nucleic acid-bound beads were washed twice with 80% ethanol. The ligated tRNA was then eluted from the beads using 23μl of nuclease-free H_2_O. The second ligation to the RMX motor adapter was performed using the ligation 1 product, 8 μl of 5× Quick Ligase Buffer, 6 μl of RMX, and 3 μl of T4 DNA ligase (2 000 000 units/ml) in a total volume of 40 μl for 30 min at room temperature. The ligation 2 product was bead purified with 1.5× (60 μl) RNA Clean XP beads. The bead purification, elution of the library, flow cell priming and loading followed the SQK-RNA002 protocol. All sequencing was done on the MinION platform.

#### Ligation with longer splints

With the goal of enabling current-based analysis of tRNA, long splint adapters were employed. The long splints consisted of a 120 nt RNA 5′ oligonucleotide which hybridized to the 3′NCCA end of tRNA and a common 60 nt strand composed of 46 RNA and 14 DNA nucleotides that abutted the 3′ end of the tRNA. Ligation conditions were similar to the standard adapters with the following modifications. Following the first ligation the bead cleanup was performed with 1× RNA Clean XP beads and washed with 70% ethanol. Following the second ligation, 0.8× rather than 1.5× bead concentration was used.

#### IVT tRNA construction and Nanopore sequencing

The DNA oligonucleotides for the IVT constructs were purchased from IDT. Their sequences are shown in Supplementary Table S2. Yeast IVT tRNAs were generated using the HiScribe T7 Quick High Yield RNA Synthesis Kit (NEB, E2050) similar to that done for *E. coli* tRNA^Ala(UGC)^ (5), but without gel purification. Two DNA oligonucleotides were used to make each IVT construct. A common 27 nt T7-promoter containing strand was used for all IVT tRNAs (Supplementary Table S2). For each of 42 tRNA isoacceptor types, a template strand was designed to hybridize with the common 27-nt adapter and to encode a transcript that included the 18 nt 5′ RNA adapter sequences followed by the tRNA, followed by the 6 nt 3′ RNA adapter sequences followed by a 3′ 14 nt polyA tail (Supplementary Table S2). The sequence used for each isoacceptor was based on the sequences in GtRNAdb (gtRNAdb.ucsc.edu) (21). For tRNAs with multiple isodecoders, we selected the sequence with greatest gene copy number as the representative isoacceptor. 200 pmol of the common 27mer and 50 pmol of the template for each of the 42 tRNA IVT constructs were hybridized separately in 1× TNE (10 mM Tris, pH 8.0, 50 mM NaCl, 1 mM EDTA) by heating to 75°C for 1min and slowly cooling to room temperature. One microliter of each of the 42 hybrids was pooled, and 7 μl of this stock was used for the IVT reaction. The IVT reaction was run for 16 h at 37°C, DNase I treated for 30 min at 37°C, cleaned up with 1.3× RNA Clean XP beads and eluted in 20 μl nuclease-free water. One microliter (934 ng total, roughly 25 ng for each IVT construct) of the reaction was sequenced following the SQK-RNA002 protocol for mRNA sequencing. 1.3× and 1× magnetic bead volumes were used for cleanup following the RTA and RMX ligations, respectively.

### Bioinformatic methods

Ionic current files were basecalled with Guppy v3.0.3 and aligned with BWA-MEM (parameters ‘-W 13 -k 6 -x ont2d’) to a custom BY4741 strain yeast isoacceptor reference generated using tRNAscan-SE (Supplementary Table S5) (22–25). Each isoacceptor reference also included the corresponding adaptor sequences (Supplementary Table S2). The FASTQ files were concatenated and processed to convert all ‘U’ nucleotide calls to ‘T’. Alignments were then filtered to a mapping quality score of greater than one (Q1) using SAMtools (26). Alignment and error models (expectation maximization, or EM) for each experimental condition were generated using marginAlign. This program optimizes Nanopore read alignments by estimating true error rates using a Hidden Markov Model (HMM) (5,27). Calculation of posterior probabilities was done with the subprogram marginCaller (parameter ‘–threshold 0’) (5,27). marginCaller calls single nucleotide substitutions in Nanopore data by using the alignment model estimated by marginAlign. It does so by using a marginalization technique that examines multiple possible alignments between a read and reference sequence. We employed marginCaller for tRNA by modeling modification-associated miscalls as single nucleotide substitutions. The IVT alignment model was used for the ‘error model’ in all experiments. This model incorporates mismatch, insertion and deletion information. The resulting posterior probabilities were visualized in heatmaps with matplotlib (28). A detailed protocol for generating heatmaps can be found on GitHub at https://github.com/nanoniki/tRNA-heatmap-generator. Other general alignment statistics were calculated with SAMtools and marginStats (Supplementary Figure S1).

The scikit-learn SVM package was used for machine learning of T-loop miscall error profiles under different genetic conditions (wild-type, *trm2*Δ, *pus4*Δ, *trm6*Δ, and IVT). Read IDs and basecalls from a 9-mer window, consisting of the T-loop (7-mer) and two adjacent up (G) and downstream (C) positions, were extracted from Q1 filtered alignment files. The T-loop basecall data were then split in a 60:20:20 ratio of training, testing, and withheld sets, respectively. Two methods of classification were utilized on these sets; global and isoacceptor specific. Under global classification, training sets consisted of reads from all isoacceptors known to have the modification being assessed (14). Under isoacceptor classification, training sets consisted of reads from only one isoacceptor at a time. For each condition, training reads were labeled as positive for the presence of a certain modification or negative for the presence of that modification depending on the literature, and testing reads were labeled based on qualitative heat map derived predictions. The prediction power of the models was assessed by calculating accuracy, ROI, and F1 score (Supplementary Table S3). More details on the SVM classifier for tRNA sequence data can be found on GitHub at https://github.com/nanoniki/tloop-training.

The marginCaller default posterior probability threshold to make an alternative nucleotide call is 0.3 (30%). However, marginCaller thresholds are only applied to a specific alternative nucleotide (27). Modifications do not always make consistent alternative nucleotide calls (e.g. the Ψ_55_ U-to-C miscall), so it is best to assess their miscall impact through the complementary probability to the reference (any alternative, also referred to as ‘mismatch probability’).


\begin{equation*}{\mathrm{P}}\left( {{\mathrm{A^{\prime}}}} \right){\mathrm{ = 1 - P(A)}}\end{equation*}

To determine this mismatch probability, we first extracted a random subset of 120 reads per tRNA isoacceptor from wild-type and IVT alignments. These subsets were run through marginCaller, and then posterior probabilities from positions affected by T-loop modifications were examined. In wild-type, if the mismatch probability was ≥0.3, those tRNAs were considered to have statistically robust miscalls. IVT results served as a negative control, as none of the mismatch probabilities from unmodified sequences met or exceeded the cutoff requirements (Supplementary Figures 2A, B).

### Global ribonucleoside modification profiling by LC–MS/MS

The RNA modification abundance of yeast total tRNA was quantified using a highly multiplexed targeted ribonucleoside LC–MS/MS assay as previously described (29). Briefly, total tRNA (100 ng) was degraded to monoribonucleosides using a two-step enzymatic digestion. The RNA was first hydrolyzed to ribonucleotide monophosphates using 300 U/μg nuclease P1 (NEB, 100 000 U/ml) overnight at 37°C in 100 mM ammonium acetate (pH 5.5) and 100 μM ZnSO_4_. Following, the nucleotides were dephosphorylated using 50 U/μg bacterial alkaline phosphatase (Invitrogen, 150 U/μl) for 5 h at 37°C in 100 mM ammonium bicarbonate (pH 8.1) and 100 μM ZnSO_4_. Prior to use, the enzymes were buffer-exchanged into their corresponding reaction buffers using a BioRad Micro Bio-Spin 6 size exclusion spin column to remove glycerol and other ion suppressing contaminates in the enzyme storage buffers. After the reactions, the samples were lyophilized and resuspended in 10 μl of water containing 40 nM ^15^N_4_-inosine internal standard.

The resulting ribonucleosides were separated by reversed phase chromatography and quantified using multiple reaction monitoring on a triple quadrupole mass spectrometer as previously described (30).

### Western blot

The western blot showing expression of Pus4-R286K and wild type Pus4 from strains with a C-terminal HA tag on Pus4 was created by separating protein prepared using trichloroacetic acid lysis on a 4–15% polyacrylamide gel in 1× SDS buffer. Protein was transferred to a nitrocellulose membrane, blocked in a 5% milk solution in 1× PBS-T, and probed for Pus4 using a 1:500 dilution of Invitrogen rabbit polyclonal anti-HA antibody (SG77; 71–5500; Lot XH355482) as a primary antibody, and a 1:1000 dilution of Sigma goat anti-rabbit HRP conjugated antibody (A6154; Lot SLCD6835) as the secondary antibody. The blot was probed for histone H3 as a loading control using a 1:2000 dilution of Abcam rabbit anti-H3 antibody (ab1791; Lot GR3297878-1) as the primary antibody and the same anti-rabbit secondary antibody. All antibodies were diluted in 5% milk solutions in 1× PBS-T and each incubation in antibody was followed by three washes in 1× PBS-T. The blot was imaged on an Amersham Typhoon using Bio-Rad Clarity Western ECL Substrate.

## Results

### Direct sequencing of yeast tRNAs

We sequenced the 42 tRNA isoacceptors—41 elongator tRNAs and 1 initiator tRNA—from the budding yeast cytosol using Nanopore DRS. By modifying the strategy we previously implemented for sequencing *E. coli* tRNA (5) (see Materials and methods), we isolated ∼60–85 nt long RNA molecules from 100 μg of total RNA using PAGE. The resulting RNA pool was then ligated to oligonucleotide splint adaptors designed to anneal and ligate with mature tRNA NCCA-3′ ends. This product was subsequently ligated to the Oxford Nanopore Technologies (ONT) RNA sequencing adapter (Figure 1B).

The adapted tRNA molecules were sequenced using ONT MinION R9.4.1 flow cells and Nanopore SQK-RNA002 kits. A representative tRNA ionic current trace is shown in Figure 1C. Segments corresponding to the adapters and the tRNA molecule were distinguishable because the ONT adapters, and part of the tRNA splint adapters (both 3′ and 5′), are composed of deoxyribonucleotides which have different amplitudes and translocation times than ribonucleotides. Conversion of ionic current patterns to nucleotide sequence base calls was performed using ONT Guppy software (version 3.0.3) (Figure 1D, E). This earlier Guppy version was chosen because it can infer sequence information from ionic current for short RNA molecules better than more recent versions (5). Each tRNA DRS experiment yielded between 22 500 and 383 000 RNA reads per flow cell (Supplementary Table S4). The range in reads per flow cell was likely due to a combination of variation in tRNA adaptation, library preparation, and flow cell performance. We aligned reads to a curated set of yeast tRNA isoacceptor sequences (Supplementary Table S5) using the BWA-MEM aligner (24).

Each yeast strain derived tRNA DRS experiment yielded between 6000 and 200 000 MAPQ1-aligned tRNA reads per flow cell (Supplementary Table S4). RNA reads that failed to align were likely due to: (i) inadequate training of the Nanopore base caller for short RNA molecules; (ii) the overall 86% median accuracy of the RNA base caller and (iii) constraints in the alignment software when dealing with short, error-prone reads. The median alignment identity observed across all 42 tRNA isoacceptors was 83%, which is lower than the 90% median identity observed for biological poly(A) RNA (31). This was expected because tRNA has a much higher abundance and density of modifications compared to mRNA. In contrast, the IVT total tRNA DRS experiment yielded ∼500 000 MAPQ1-aligned reads, 91% of which aligned to full-length molecules, reflective of their complete lack of modifications (Supplementary Table S4). Importantly, most aligned tRNA reads from yeast cells (83–93%) were full-length sequences (Supplementary Table S4). Among the four replicates of wild-type *S. cerevisiae* tRNA, the lowest number of aligned reads was between 3 and 106 for tRNA^Ser(UGA)^, and the highest number ranged from 1170 to 12 237 for tRNA^Leu(CAA)^ (Supplementary Table S6, Supplementary Table S7). A representative alignment of Nanopore reads for biological tRNA^Pro(UGG)^ is shown in Figure 1E. The ‘reference match probability’ for each of the tRNA positions are shown in the heatmap compiled in Figure 1F. The values at each tRNA position in the heat map indicate the probability that a Nanopore base call matched the canonical unmodified reference nucleotide, measured using an error model based on mismatches, insertions, and deletions, derived from both our IVT DRS data and other parameters (see Materials and methods). It is important to note that these values are probabilities, and do not represent quantitative measurements of the presence of an unmodified base versus an alternative.

### DRS detects conserved pseudouridine modifications in the T-loop of yeast tRNAs

**Figure 2.**
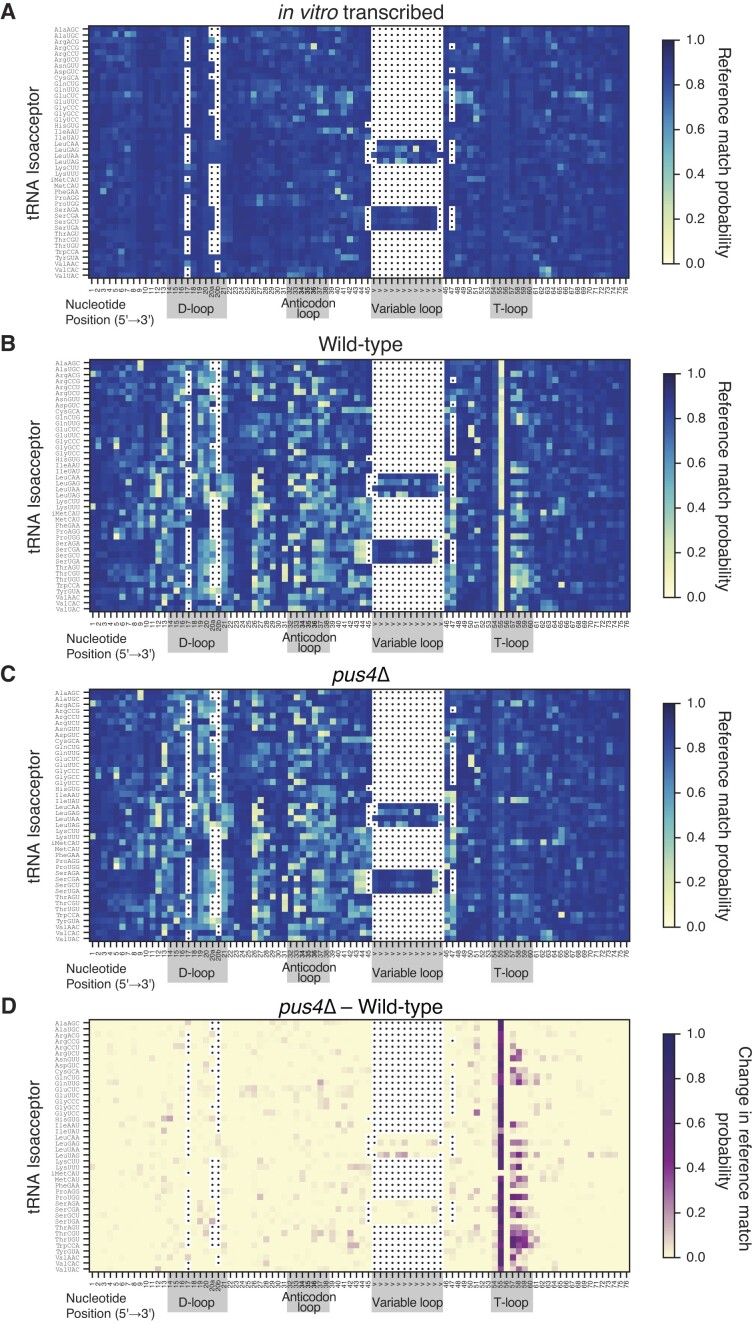
Heatmaps representing comprehensive alignments of 42 *S. cerevisiae* cytosolic isoacceptors exhibit miscalls coincident with modified positions in the T-loop and elsewhere. (**A**) Heatmap representing *in vitro* transcribed (IVT) tRNA sequences of 42 *S. cerevisiae* cytosolic isoacceptors. A higher reference match probability (dark blue) corresponds to positions where the base-called nucleotide more frequently concurs with the reference nucleotide given the alignment, and a lower reference match probability (light yellow) corresponds to positions where the base-called nucleotide more frequently disagrees with the reference nucleotide. Sequences were aligned by the D-loop, anticodon loop, variable loop, and T-loop—highlighted with grey boxes, with anticodon positions in bold type; white boxes with dots indicate gaps—following a conventional tRNA base numbering scheme and based on the sequence alignment in Supplementary Figure S3. Note that tRNA^His(GUG)^ reads were aligned beginning with position 1 for simplicity, however note that this tRNA contains a non-templated G added at 5′ end in the ‘-1’ position (36). (**B**) Wild-type aligned isoacceptors. Otherwise as described above. (**C**) *pus4*Δ aligned isoacceptors. Otherwise as described above. (**D**) The change in reference match probabilities between *pus4*Δ and wild-type aligned isoacceptors. As described above except that dark purple squares correspond to large differences in basecalls between tRNAs in the two strains and yellow squares show basecalls that are not different between the two strains.

To visualize base miscalls in the alignments of DRS data for yeast tRNA isoacceptors, we assembled composite heatmaps using reference match probabilities. Our heatmaps display sequences using a conventional tRNA base numbering scheme (33), aligning base position within the D-loop, anticodon loop, variable loop and T-loop, guided by our manually curated sequence alignment of all 42 isoacceptors (Supplementary Figure S3). The heatmap for IVT tRNA sequences that accounts for mismatches that may result from certain canonical nucleotide sequence contexts shows a majority of dark blue squares and limited number of yellow/lighter-toned squares (Figure 2A). This reflects the generally high reference match probability values obtained from sequencing unmodified RNAs, as expected. For tRNA obtained from wild-type yeast cells, many positions align as expected, yielding dark blue squares. However, in contrast to the IVT data, a significant subset of positions across all tRNAs mismatch the reference nucleotide, yielding yellow-toned squares which indicate likely sites of modification (Figure 2B). The most distinct of these miscall patterns was observed within the T-loop.

Nucleotides at position 55 of the T-loop were the most consistently miscalled in 41 of the 42 isoacceptors. The one exception was tRNA^iMet^, the only yeast tRNA known to lack a pseudouridine at that position (34). Therefore we reasoned that this miscall pattern was due to a highly conserved pseudouridine in tRNA (Ψ_55_) (35), as seen in our previously documented Nanopore DRS data for *E. coli* tRNA (5). To test this, we sequenced tRNAs from a yeast strain (*pus4*Δ) that lacks the enzyme Pus4, that catalyzes conversion of U_55_ to Ψ_55_ (Figure 2C). Miscalls at this position in this *pus4*Δ strain were substantially reduced. Other miscall patterns across the T-loop were also altered for some isoacceptors, suggesting that additional Pus4-dependent base miscalls occur at positions 57–59 (Figure 2D). Miscall patterns outside the T-loop were highly similar between the two strains, suggesting that Pus4 pseudouridylation did not influence other (miscall-generating) modifications elsewhere in any tRNAs.

**Figure 3.**
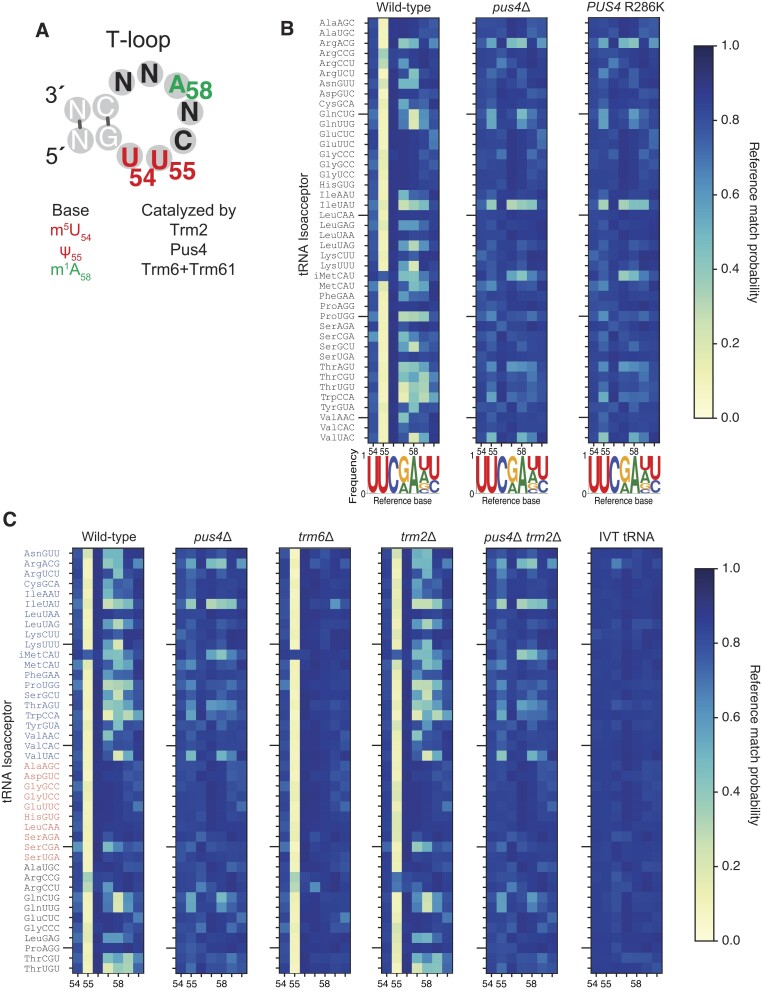
Reference match probabilities identify changes corresponding to modifications in the T-loop across 42 cytosolic isoacceptor tRNAs. (**A**) Illustration of a generic yeast tRNA T-loop, including its three possible chemical modifications and the names of the enzymes that catalyze them. (**B**) Reference match probability heat maps of aligned T-loop sequences across 42 cytosolic isoacceptor tRNAs, in wild-type, *pus4*Δ and *PUS4* R286K strains. Conventional tRNA position numbers of modifications (m^5^U_54_; Ψ_55_; m^1^A_58_) are shown below each plot. In the sequence logos below, the height of each unmodified nucleotide letter matches its frequency in shown tRNA sequences. (Note that for tRNA^iMet^, in contrast to other 41 isoacceptors, positions 54 and 60 are adenosines, barely visible in the logo.) (**C**) Reference match probability heat maps for tRNAs purified from wild-type, *pus4*Δ, *trm6*Δ, *trm2*Δ, *pus4*Δ*trm2*Δ strains and *in vitro* transcribed (IVT) control sequences. tRNAs are ordered differently than in previous panels. tRNAs in blue letters are those annotated in Modomics (14) to contain m^1^A in the T-loop; tRNAs in red letters are those annotated in Modomics to not contain m^1^A in the T-loop; tRNAs in black letters are those not included in the current version of Modomics.

The T-loop is seven nucleotides in all yeast cytosolic tRNAs, with some positions being conserved and others variable (Figure 3A, B, Supplementary Figure S3, Supplementary Table S8). We focused our further analysis on the systematic miscall patterns in T-loops, with heatmaps beginning at the first nucleotide at the 5′-end of the T-loops of all isoacceptors (Figure 3B). This reproduced the miscall pattern observed in Figure 2 showing that *pus4*Δ lacks a miscall at position 55 and exhibits alteration in miscall patterns near the 3′-end of the T-loop.

To verify that the catalytic activity of Pus4 was responsible for the loss of Ψ_55_ and its associated miscall patterns, and not some other role of this enzyme, we generated a yeast strain that expresses a Pus4 protein containing an R286K mutation predicted to inactivate its catalytic activity. The mutation was based on homology to TruB, the bacterial homolog of Pus4 (37) (Supplementary Figure S4). This *PUS4* R286K strain produced a miscall pattern nearly identical to cells missing the *PUS4* gene entirely (Figure 3B). Therefore, we concluded that the miscall patterns in the T-loop were due to changes in tRNA modification status (U_55_/Ψ_55_), and not other activities of the enzyme itself.

### DRS detects conserved 1-methyladenosine modifications in the T-loop of yeast tRNAs

While the miscall pattern observed at position 55 was consistent with extensive prior literature documenting pseudouridine at that position in most tRNAs in nature (14,38,39), we considered other possible explanations for the additional miscalls in positions 57–59. Nanopore sequencing errors arising from sequence context are unlikely to be responsible because wild-type, *pus4*Δ, and IVT tRNA isoacceptors have the same canonical sequences, and IVT sequences did not produce strong miscalls anywhere in the T-loops of all 42 isoacceptors (Figure 3C). We next examined if pseudouridine might lead to base miscalls at the level of nucleotide kmers and not individual nucleotides, as previously observed for m^7^G in 16S rRNA in Nanopore data (12). However, the initiator methionine tRNA is not pseudouridylated at position 55 and miscalls proximal to the 3′ side of the T-loop were still observed for this tRNA in both WT and *pus4*Δ data (Figure 3B). Furthermore, prior studies of Ψ using DRS in other types of RNAs have consistently observed single nucleotide miscalls matching known modified positions (7–9,12,40,41).

A biochemical explanation thus seemed most likely. Since the miscall pattern was consistently proximal to position 58 (the 5th position of the T-loop), we reasoned that it might be due to the presence of a 1-methyladenosine modification at position 58 (m^1^A_58_) (42). We grouped the tRNA isoacceptors in our T-loop heat map based on prior evidence of m^1^A_58_ cataloged by Modomics, the widely used RNA modifications annotation database (14) (Figure 3C). In general, miscalls proximal to position 58 reported by Nanopore correlated well with Modomics annotations specifying the presence of m^1^A_58_ modification (tRNAs in blue font) or its absence (tRNAs in red font). Yeast tRNA isoacceptors with unknown m^1^A_58_ status, according to Modomics, demonstrated a mixture of miscall patterns, with some predicting the presence of m^1^A_58_ and others predicting its absence (Figure 3C, wild-type, black font).

To determine the source of miscalls proximal to position 58, we compared basecalls from wild-type cells to basecalls from a yeast strain lacking the gene encoding the enzyme Trm6 (43). Trm6 forms a methyltransferase complex with Trm61 to methylate the conserved A_58_ (44). The gene *TRM6* is essential due to the fact that tRNAs lacking m^1^A_58_ become destabilized and subsequently degraded primarily by the nuclear RNA surveillance pathway (45–47), causing lethality. This defect can be suppressed via constitutive overexpression of one of four yeast iMet tRNA gene copies, whose encoded tRNAs are normally methylated in wild-type cells by this complex to promote their structural stability (43). We sequenced this strain—*trm6*Δ with a high-copy plasmid expressing the iMet tRNA gene *IMT4*, henceforth referred to as ‘*trm6*Δ’—in order to determine whether m^1^A_58_ was responsible for the pattern of miscalls at positions 57–59 in wild-type yeast. We observed that the *trm6*Δ strain produced no miscalls at these positions in the T-loop of tRNAs that were previously reported to have m^1^A_58_ (Figure 3C). This strongly suggested that these miscalls observed in wild-type cells were due to m^1^A catalyzed by the Trm6/Trm61 methyltransferase complex. Importantly, the base miscall pattern observed at position 55 that results from pseudouridylation in wild-type yeast was maintained in *trm6*Δ cells. We also observed a similar pattern of base miscalls for a yeast strain lacking *TRM61*, that also inactivates the methyltransferase activity (43) (Supplementary Figure S5). These data suggested that for many isoacceptors, pseudouridylation may precede adenosine methylation, and that m^1^A_58_ modification does not appreciably influence Pus4 activity.

### Agreement between m^1^A_58_ annotations by DRS and other methods

We were able to detect m^1^A_58_ in 24 of 42 cytosolic tRNAs via the changes in mismatch probabilities at positions 57–59 between wild-type and *trm6*Δ (Supplementary Table S9, Supplementary Table S10). We made these predictions using a ≥0.3 posterior probability cutoff, the default used by the program marginAlign (Materials and methods). We note that even above this cutoff, while there was variation in the value of reference match probabilities at these positions in different isoacceptors, it is important to restate that these are probability values and not quantitative measurements of the modification ‘stoichiometry’, or the fraction of molecules of each isoacceptor that contain m^1^A_58_. DRS corroborated 18 of the 21 tRNAs stated to contain m^1^A_58_ by Modomics. Three tRNAs that did not match Modomics m^1^A_58_ predictions were tRNA^Leu(UAA)^, tRNA^Lys(CUU)^, and tRNA^Val(CAC)^. These tRNAs were also not predicted to have m^1^A_58_ by ARM-seq, a reverse transcriptase (RT)-based sequencing method (48). Another RT-based method, mim-tRNAseq, previously revealed modest base misincorporation at position 58 for these three tRNAs during reverse transcription—corroborating Modomics status (4). These differences could be due to sample or biological variation between experiments and differences in the limit of detection between different technologies. The recent Nanopore DRS-based study by Lucas *et al.* also associated base miscalls with T-loop modifications and observed overall agreement with Modomics annotations, however it did not perform analysis to make specific assignments for which isoacceptors were predicted to have m^1^A_58_, nor did it provide a mutant lacking m^1^A_58_ for comparison (6).

Modomics specifies that 10 yeast tRNAs are *not* modified at position 58. Our DRS miscall-based predictions agreed for 9 of these 10 tRNA isoacceptors (Supplementary Table S9). The exception, tRNA^Ser(CGA)^, was also reported to contain m^1^A_58_ by ARM-seq and mim-tRNAseq (4,48). Modomics currently does not report any chemical modifications for 11 additional tRNA isoacceptors—not only at position 58, but at any position—due to lack of prior experimental data. Using the ≥0.3 posterior probability cutoff from wild-type data (and excluding cases where this threshold was also exceeded in *trm6*Δ data), we predicted the presence of m^1^A_58_ on the Gln(CUG), Gln(UUG), Leu(GAG), Thr(CGU) and Thr(UGU) isoacceptors, and absence of m^1^A_58_ on Ala(UGC), Arg(CCG), Arg(CCU), Glu(CUC), Gly(CCC) and Pro(AGG). ARM-seq and mim-tRNAseq corroborate all 11 of these DRS-based predictions (Supplementary Table S9) (4,48).

### 5-methyluridine does not yield robust base miscalls in the T-loop

A recent study profiling tRNAs by DRS reported that 5-methyluridine (m^5^U) led to a significant amount of base miscalls in the first position of yeast tRNA T-loops (6). We performed a m^5^U-specific mismatch analysis using our data, but accounted for three additional parameters that were not accounted for in that study. First, we used a genetic control in which the modification was eliminated (*trm2*Δ). Second, we addressed the potential miscall signal ‘bleed-through’ from proximal modifications. Third, we accounted for DRS sequencing error by using IVT tRNA controls to establish a canonical RNA error model (see Materials and methods) (27). We performed this analysis using a randomly downsampled set of 120 reads per tRNA isoacceptor. At position 54, the median miscall probability was 0.16 in aligned reads from IVT data (Supplementary Table S10). For wild-type data, the median miscall probability at position 54 was 0.21 (Supplementary Table S10). For *trm2*Δ and *pus4*Δ strains, the median miscall probability at position 54 was 0.16 and 0.2, respectively (Supplementary Table S10). We argue that these base miscalls at position 54 cannot be confidently attributed to the m^5^U_54_ modification because they fell within the bounds of DRS error, or a mismatch probability of <0.3. Software improvements for base calling and ionic current analysis will be required for a better identification of m^5^U_54_ signatures in tRNA.

Even though m^5^U_54_ did not result in a miscall pattern in Nanopore DRS, we reasoned that analysis of *trm2*Δ mutants lacking m^5^U_54_ could be used to investigate if this modification had an influence on catalysis of Ψ_55_ or m^1^A_58_. Loss of m^5^U_54_ had little-to-no impact on the m^1^A_58_ base miscall signal (Figure 3C). There were no tRNA isoacceptors that had noticeably altered reference match probabilities proximal to position 58 in *trm2*Δ strains—i.e. the m^1^A_58_ status in *trm2*Δ strains appears unchanged for all isoacceptors. If biologically relevant changes in m^1^A_58_ did occur depending on m^5^U_54_ status, they were under our threshold of detection comparing *trm2*Δ cells to wild-type using miscalls. This potential limitation is a possibility for at least one case, as previous studies have established the influence of m^5^U_54_ on catalysis of m^1^A_58_ in yeast tRNA^(Phe)GAA^ (19,63).

Loss of Trm2 alone did not appear to generally reduce the presence of Ψ_55_ across tRNA isoacceptors either (Figure 3C). Two possible exceptions, however, were tRNA^Arg(CCG)^ and tRNA^Arg(CCU)^ which have a reduced base mismatch probability at position 55 compared to wild-type cells. This suggests that they may have had lower levels of pseudouridylation when m^5^U_54_ is absent.

### Ψ_55_ promotes m^1^A_58_ installation in certain tRNAs

Many groups have reported evidence of coordination of RNA modifications in tRNAs, also referred to as modification ‘circuits’ (6,19,49–62). We reasoned that this phenomenon should be evident in our data because direct RNA sequencing can simultaneously profile multiple modifications on individual, full-length strands (e.g. Ψ_55_ and m^1^A_58_). Both m^5^U_54_ and Ψ_55_ have been shown to influence addition of m^1^A_58_ in yeast tRNA^(Phe)GAA^, using purified enzymes and synthetic tRNAs (19,63). To assess this possibility across the other 41 yeast tRNA isoacceptors, we analyzed mutants lacking each of the three T-loop modifying enzymes (Pus4, Trm2 and Trm6). Elimination of Ψ_55_ lead to a strong reduction in the base miscalls attributed to Trm6 catalysis (Figure 3C). Ordering tRNAs by m^1^A_58_ status helped identify at least four classes of tRNAs based on the role of Ψ_55_ in promoting m^1^A_58_ modification (Supplementary Table S11). Class I contains a single example: the iMet tRNA, which does not have Ψ_55_ but does have m^1^A_58_. Therefore its m^1^A_58_ is added independent of Ψ_55_. Class II are tRNAs that have Ψ_55_ but lack m^1^A_58_. Therefore Ψ_55_ also has no influence on them being substrates for the Trm6/Trm61 methyltransferase complex. Class III are examples similar to tRNA^Phe(GAA)^ that have both Ψ_55_ and m^1^A_58_, and for which addition of Ψ_55_ appears to strongly promote m^1^A_58_ catalysis. This was evident by the fact that when these tRNAs lose Ψ_55_, they also have significantly diminished m^1^A_58_ levels (Figure 3C, *pus4*Δ). Class IV are tRNAs with both Ψ_55_ and m^1^A_58_, but in which loss of Ψ_55_ does not appreciably reduce m^1^A_58_. These include Arg(ACG), Ile(UAU), Thr(AGU) and Val(UAC) tRNAs. Since deletion of Pus4 did not eliminate the U_55_-associated miscall entirely for all isoacceptors—most notably for Arg(ACG), Gln (CUG), Gln(UUG), Ile(UAU) and Val(UAC)—we cannot yet exclude the possibility that in the absence of Pus4, a different pseudouridine synthase—or other uridine-modifying enzyme—modifies U_55_, in turn promoting m^1^A_58_ in some of these Class IV tRNAs. Analysis of double-knockouts of Pus4 along with other pseudouridine synthase enzymes, in future work, may reveal more about this possibility. Since we report 19 isoacceptors with reduced levels of m^1^A_58_ upon removal of Ψ_55_ under these conditions, we note that any phenotypes associated with removal of *PUS4* (e.g. *pus4*Δ strain) or inactivation of its catalytic activity (e.g. *PUS4* R286K), are potentially a consequence of both loss of Ψ_55_ in all isoacceptors *and* reduction of m^1^A_58_ in most isoacceptors that normally contain it.

It is important to note that the Class assignments here are based on data in which cells are growing exponentially, and thus could vary under other growth conditions (see below). And while it is tempting to examine the sequences or other features of the tRNAs that fall into the Class III and Class IV tRNAs, to arrive at models that might explain specificity, we note the complexity observed from the recent dissection of sequence features that promote methylation for even a single example, yeast tRNA^Phe(GAA)^, that is involved in this circuit (63). A previous study also evaluated T-loop bases that promote methylation of a eubacterial tRNA^Phe^ by its corresponding methyltransferase (64), however the preferred recognition sequence is not common among the Class III and Class IV tRNAs described here, indicating additional determinants.

Because previous reports indicated independent contributions of Ψ_55_ and m^5^U_54_ on m^1^A_58_ addition, we constructed *pus4*Δ*trm2*Δ. This double mutant lacks both Ψ_55_ and m^5^U_54_ in the T-loop of tRNA isoacceptors. The base miscall pattern proximal to position 58 generated by this mutant very closely resembled that of the *pus4*Δ strain (Figure 3C).

### Nanopore DRS reveals increased m^1^A_58_ frequency in some tRNAs upon nutrient depletion

**Figure 4.**
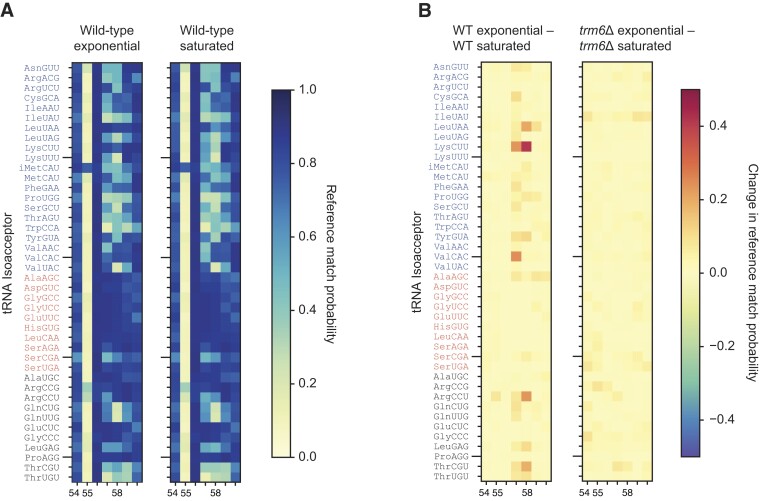
Nanopore reveals increased m^1^A_58_ frequency on some tRNAs upon nutrient depletion. (**A**) Reference match probability heat maps of the T-loop in wild-type cells growing exponentially (reprinted from Figure 3C) or to saturation. Otherwise as displayed in Figure 3C. (**B**) Bi-directional, differential reference match probability heat maps of the T-loop in wild-type or *trm6*Δ cells, comparing changes observed between exponentially growing and saturated cells. A positive change in reference match probability (red squares) indicates an increase in base miscalls during saturated phase. Comparison between heat maps indicates that several isoacceptors, most prominently tRNA^Lys(CUU)^, had increases in m^1^A_58_ during saturation that was catalyzed by Trm6.

All data reported thus far were from yeast strains harvested during exponential growth in nutrient-rich media when the growth rate is maximal. To investigate if a different growth condition could affect T-loop modifications, we grew wild-type yeast cultures to saturation. Under these conditions, growth essentially stops as nutrients become limited. While base miscall patterns remained unchanged for most tRNAs in this sample (Figure 4A), some had noticeable differences. In particular, tRNA^Lys(CUU)^ showed a newly appearing pattern of base miscalls proximal to A_58_ that was absent from this tRNA in exponentially growing cells (Figure 4A). The miscall pattern was consistent with the miscalls present across other tRNAs that have m^1^A_58_. To test whether it was m^1^A_58_ that caused this change, we grew *trm6*Δ yeast to saturation and sequenced their tRNAs. In this strain, the miscall cloud proximal to A_58_ disappeared, confirming that the change was due to an increase in m^1^A_58_ catalyzed by Trm6 (Figure 4B). We observed a similar pattern for tRNA^Leu(UAA)^ and tRNA^Val(CAC)^. Our annotation of these three tRNAs gaining an m^1^A_58_ upon growth saturation was based on the same metric used for our assignments in exponentially growing cells—a reference nucleotide posterior probability below 0.7 at positions 57, 58 or 59. While tRNA^Arg(CCU)^ also appeared to gain a signal consistent with m^1^A_58_ upon saturation in wild-type cells, the reference nucleotide posterior probability proximal to position 58 also fell <0.7 in wild-type cells growing exponentially as well as in *trm6*Δ cells grown to saturation. Thus we cannot confidentially attribute this change to an increase in m^1^A_58_. In summary, while tRNA^Leu(UAA)^, tRNA^Lys(CUU)^ and tRNA^Val(CAC)^ did not have observable base miscalls around position 58 in the cells grown exponentially—we categorized them above as ‘Class II’ under those conditions—we did document their *TRM6*-dependent base miscalls after they reached saturation. Future DRS analysis of a *pus4*Δ strain grown to saturation may reveal whether, under these conditions, these three isoacceptors fall into Class III or Class IV in regard to whether their Ψ_55_ influences catalysis of m^1^A_58_.

We hypothesize that the proportion of these tRNAs that contain m^1^A_58_ varies under different growth conditions. This can range from being absent in exponentially growing cells to abundant in saturated cells. This may also explain variation in prior annotations for these specific isoacceptors. For example, depending on how cells are grown, the m^1^A_58_ signal may be more pronounced, while in other conditions the magnitude of the miscall is under the minimum threshold. Previous studies report that m^1^A_58_ plays a role in stabilizing tRNA structure (42,63,65,66), and can prevent the degradation of the yeast iMet(CAU) tRNA (43). Additionally, in mammalian cell lines it has been shown that m^1^A_58_ levels respond to glucose availability (67). Thus, it is possible that in some tRNAs, the stress experienced by cells grown to saturation leads to an increase in m^1^A_58_.

### Mass spectrometry measurements confirm and extend evidence of interrelated changes to tRNA modifications in mutant strains

To validate if the Nanopore sequencing miscalls in the T-loop were caused by the presence of Ψ_55_ and m^1^A_58_, we used LC-MS/MS as an orthogonal method to measure global modified ribonucleoside abundance. We purified total yeast tRNA from wild-type, *pus4*Δ, *trm6*Δ, *trm2*Δ and *pus4*Δ*trm2*Δ yeast strains and then converted the complete tRNA molecules to mono-ribonucleosides using a two-step enzymatic digestion (29). The resulting ribonucleosides were separated by reversed phase chromatography and quantified using multiple reaction monitoring on a triple quadrupole mass spectrometer. This method provides a broad quantitative picture of how the tRNA modification landscape is altered among different enzyme knockouts (Supplementary Figure S6A). It also allowed us to determine the relative purity of our tRNA samples by analysis of modifications known to be present in *S. cerevisiae* rRNA, but not tRNA (14,68). Two such modifications, m^3^U and m^1^Ψ, were found in 0.006% and 0% of nucleosides, respectively, in our wild-type samples, verifying that our tRNA samples had minimal contaminating rRNA fragments.

**Figure 5.**
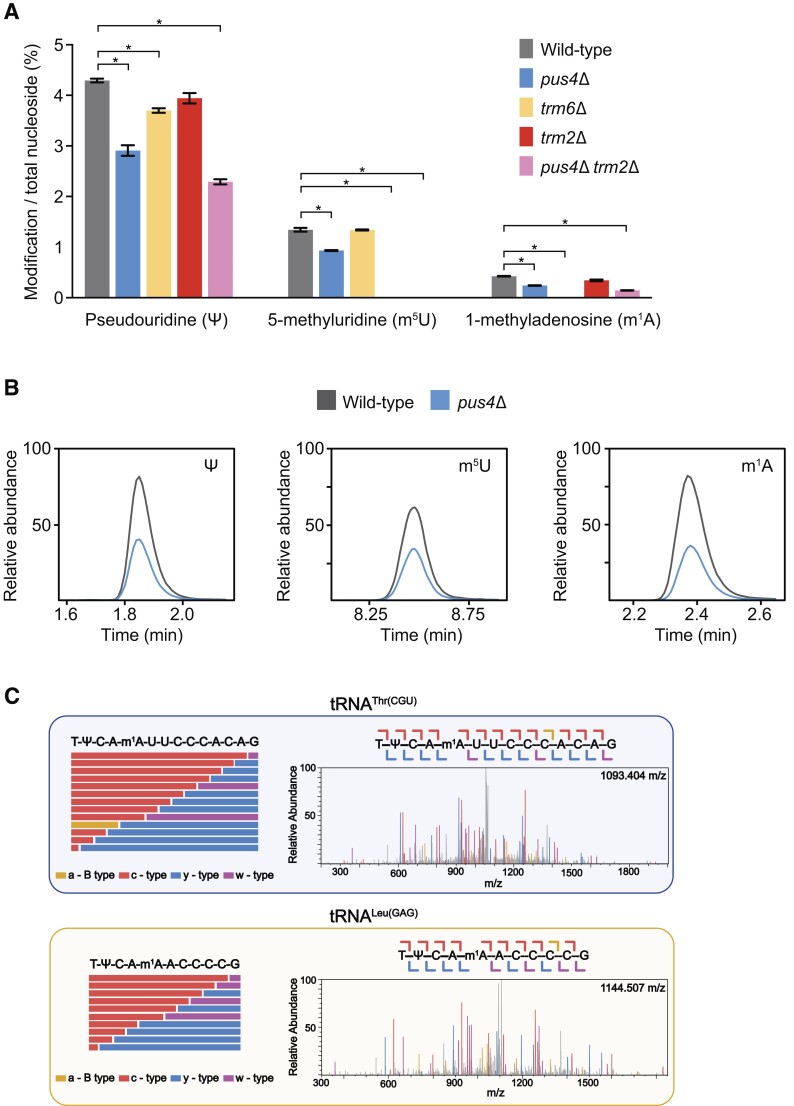
LC-MS/MS confirms DRS-based modification predictions. (**A**) Modification abundance in wild-type (grey), *pus4*Δ (blue), *trm6*Δ (gold), *trm2*Δ (red) and *pus4*Δ*trm2*Δ (pink) total tRNA quantified using LC-MS/MS ribonucleoside modification profiling. Significant changes (*P* < 0.01) are noted with an asterisk. (**B**) Extracted ion chromatograms of m^1^A and m^5^U signals in wild-type (grey) and *pus4*Δ (blue) displaying a decrease in abundance in *pus4*Δ total tRNA. (**C**) We used Collision-induced dissociation (CID) fragmentation to confirm the location of m^1^A found by DRS. CID fragments oligonucleotides at each phosphodiester backbone, resulting in ladder oligonucleotide fragments that can be detected within the MS/MS spectrum. This enables the sequencing of oligonucleotides by MS/MS, in addition to locating post-transcriptional modifications within these specific fragments. Here, we display two MS/MS fragmentation spectra and sequence coverage maps confirming that m^1^A_58_ is found within tRNA^Thr(CGU)^ and tRNA^Leu(GAG)^. (left) Sequence coverage maps displaying sequence informative MS/MS fragmentation ions for m^1^A containing oligonucleotide digestion products resulting from RNase T1 digestion of tRNA^Thr(CGU)^ and tRNA^Leu(GAG)^. (right) The respective MS/MS spectra for tRNA^Thr(CGU)^ and tRNA^Leu(GAG)^ are displayed, where each detected sequence information fragmentation ion is displayed in gold (a-B type), red (c-type), blue (y-type), and purple (w-type). Full MS/MS sequence coverage was achieved with confidence scores greater than 99% by BioPharma Finder, confirming the presence of m^1^A at the expected nucleotide position in tRNA^Thr(CGU)^ and tRNA^Leu(GAG)^. Each detected MS/MS fragmentation ion was manually inspected to confirm correct isotopic distributions. The approach, analysis and data presentation are drawn from standards for MS analysis of oligonucleotides (71–75).

In wild-type total yeast tRNA, we documented that Ψ and dihydrouridine (D) were the most abundant ribonucleosides (Figure 5A, Supplementary Figure S6B, Supplementary Table S12), consistent with previous studies (14,69,70). The amount of individual modified ribonucleosides matched expectations for each knockout: Ψ was considerably reduced in *pus4*Δ but not completely absent because other Pus enzymes can catalyze pseudouridine outside the T-loop in tRNA; m^1^A was completely eliminated from *trm6*Δ cells, confirming that the A_58_ site in the T-loop was the only position modified by this enzyme; m^5^U was completely eliminated from *trm2*Δ cells; and Ψ and m^5^U were correspondingly reduced and eliminated, respectively, from *pus4*Δ*trm2*Δ cells (Figure 5A). The Nanopore sequencing results suggested that the global reduction in Ψ in *pus4*Δ by LC–MS/MS could be attributed to the loss of Ψ_55_ across nearly if not all tRNAs. In summary, this provided direct physical and quantitative evidence that miscalls observed in DRS at position ∼55 were due to Ψ_55_ and those at positions 57–59 in the T-loop were due to m^1^A_58_.

The total ribonucleoside LC–MS/MS data provided further evidence that knockout of RNA modifying enzymes broadly altered the tRNA modification landscape within the T-loop of total yeast tRNA. For example, in *pus4*Δ cells we detected a reduction in the abundance of m^1^A by ∼40% and m^5^U by ∼30% in addition to the reduction of total Ψ by ∼30% (Figures 5A, B). For m^1^A, this corroborated the DRS data, which showed a decrease in miscalls at positions 57–59 across many of the tRNA isoacceptors. For m^5^U, this provided independent measurement of a modification that was not associated with a base miscall in DRS data, but did match previous observations of the influence of Ψ_55_ on m^5^U_54_ (19,63). We also noted combinatorial effects for the other mutants—while the modest reductions of m^1^A and Ψ in the *trm2*Δ cells did not pass our statistical significance threshold, the *pus4*Δ*trm2*Δ strain showed significant decreases in the levels of Ψ and m^1^A, in comparison with either *pus4*Δ *or trm2*Δ alone (Figure 5A). Prior reports have also demonstrated the influence of m^5^U_54_ on addition of m^1^A_58_ (19,63). Unlike m^1^A and m^5^U that are only known to occur in the T-loop, changes in Ψ levels measured here—for example in comparing the levels from *pus4*Δ versus *pus4*Δ*trm2*Δ—could involve changes in the T-loop and other positions this modification is known to occur in many tRNAs. Finally, we note that some changes in the mass spectrometry analysis of *trm6*Δ cells could be confounded by the greatly altered relative abundances of tRNAs in this mutant (Supplementary Table S7). Together, these findings provide evidence for the importance of combining DRS analysis with an orthogonal physical measurement like LC–MS/MS of total ribonucleosides to provide a comprehensive and quantitative picture of the tRNA modification landscape.

### Nanopore-based predictions of m^1^A_58_ sites are confirmed by LC–MS/MS-based direct tRNA sequencing

As described above, when considering our data from both exponentially growing as well as saturated cells, the base miscall profile associated with m^1^A_58_ was concordant with prior measurements for 30 out of 31 isoacceptors currently listed in the Modomics database. The only exception was that our DRS analyses predicted m^1^A_58_ on Ser(CGA) while Modomics did not. Both mim-tRNAseq and ARM-seq also predicted that Ser(CGA) has m^1^A_58_ (Supplementary Table S9).

For the 11 other isoacceptors not currently listed in Modomics, we used DRS miscalls to predict the status of m^1^A_58_, and then attempted to cross validate those using orthogonal methods. Comparing our data with mim-tRNAseq and ARM-seq, we noted majority agreement among the predictions (Supplementary Table S9). We then confirmed the status of the m^1^A_58_ sites by analyzing data previously collected using a ‘bottom-up’ RNA sequencing methodology that performs direct sequencing of tRNA using LC–MS/MS (30). In short, wild-type yeast total tRNA was partially digested by RNase T1 to shorter oligonucleotide fragments that could be assigned to spectra with or without a unique set of modifications. Oligonucleotides were separated by hydrophilic interaction chromatography and sequenced using high-resolution MS/MS (Supplementary Figure S6A).

Our reanalysis of this direct sequencing of wild-type yeast tRNA by LC–MS/MS confirmed the presence of m^1^A_58_ in 14 out of 21 tRNAs previously annotated to contain m^1^A_58_ in the Modomics database. Similarly, these data failed to detect m^1^A_58_ on six of the ten tRNAs reported in Modomics to lack m^1^A_58_ (Supplementary Table S9). For the 11 tRNAs lacking any prior annotations of modifications in the Modomics database, but for which our Nanopore data predicted five of them as having m^1^A_58_, the bottom-up MS-based sequencing was able to confirm two of those five contained m^1^A_58_: tRNA^Leu(GAG)^ and tRNA^Thr(CGU)^ (Figure 5C, Supplementary Table S9). The nine remaining isoacceptors were either too low in abundance to be detected by this method or did not produce unique spectra. Since this method relies on incomplete digestion of RNAs, we note that this type of data cannot provide *quantitative* measurements of modification abundance either. While our Nanopore DRS and total nucleoside MS analysis were performed on tRNA samples from identical growth conditions, those used for this bottom-up sequencing were purchased from a commercial vendor (Sigma) that perform large-scale preparations of cells/RNA. Remarkably, even these notable differences in the scale at which cells were grown still yielded highly similar assignments of m^1^A_58_ compared to Nanopore DRS (Supplementary Table S9). Overall, our comparisons demonstrate how direct RNA-sequencing by LC–MS/MS is highly complementary to Nanopore DRS, both for clarifying prior annotations as well as establishing new modification assignments in tRNAs. Application of these two methods, together with quantitative LC–MS/MS, to RNA oligonucleotide standards containing pre-defined combinations of different modifications, will provide the training data to help advance DRS toward true *quantitative* and *single molecule* analysis of modifications.

### Development of a model to predict T-loop modifications in tRNA

The heatmap representation of reference match probabilities at positions along the tRNAs provided a qualitative analysis of cumulative miscalls and their association with modifications (Figure 3). To extend these qualitative results with statistics, we employed a support vector machine (SVM) to generate a classifier whose input is a T-loop miscall profile (see Materials and methods). This could be used, for example, to generate a list of putative modification sites within the T-loop which could be verified with other methods. While tRNA modifications have been profiled extensively in yeast tRNAs, little is known about the modification landscape in nearly all other eukaryotic organisms.

The bounds for our analysis was a 9-mer window, composed of the seven nucleotide T-loop and one nucleotide upstream (guanosine) and one downstream (cytidine), as this base pair identity is conserved across all yeast cytosolic isoacceptors and its signal is likely to influence that in the terminal bases of the loop. Our training sets for the classifier consisted of: i) a ‘global’ profile representing miscall data from all 42 wild-type isoacceptors; and ii) a series of 42 independent ‘local’ profiles, representing miscall data from each individual isoacceptor (see Materials and methods). These two classes of models were trained by assigning the status for a modification as being ‘present’ or ‘absent’—a two-class classifier—based on existing Modomics annotations and our bottom-up LC–MS/MS data, but not the DRS data. We then used a reference match probability threshold of ≥0.3 on Nanopore data to make a prediction for whether a miscall could be associated with a modification (see Materials and methods). The labels in the testing set were assigned based on those predictions (Supplementary Table S3).

The classification accuracies of global models for predicting presence or absence of m^1^A_58_ and m^5^U_54_ on a tRNA isoacceptor ranged between 91–94% and between 51–57%, respectively (Supplementary Figure S7A, Supplementary Table S3). For reference, a 50% accuracy for a two-class classifier suggests that the model is unable to differentiate between them. Thus, due to our conclusions regarding the lack of miscall signal at U_54_ described above, the model did not predict the presence of m^5^U_54_. Similarly, since DRS signal indicated that 41/42 isoacceptors contain Ψ_55_, the weighted global model does not predict absence of this modification combined with m^1^A_58_. We did not train a global model for Ψ_55_ alone because of the strongly correlated dependence of m^1^A_58_ on Ψ_55_, as observed in the *pus4*Δ strain described above.

The classification accuracies of isoacceptor-specific models for predicting presence or absence of m^1^A_58_ and m^5^U_54_ ranged between 54–92% (82% overall) and 58% (58% overall), respectively (Supplementary Table S3). Accuracy values ∼50% were observed for isoacceptors predicted to lack m^1^A_58_—this was expected as the signal profile in wild-type data closely matched that in *trm6*Δ, so the classifier chose each option equally. As observed for the global model, the local m^5^U_54_ model accuracy remained poor because it could not distinguish between wild-type and the *trm2*Δ reads. The isoacceptor-specific models to test the combined absence of Ψ_55_ and m^1^A_58_ had a median accuracy of 92% (Supplementary Figure S7B). Lastly, we trained models using miscalls over the entire T-loop, comparing between wild-type and IVT tRNA data. The median accuracy for these models was 97% (global profiles) and 97% (isoacceptor-specific profiles)(Supplementary Figure S7A, B).

## Discussion

In this study, we leveraged Nanopore direct RNA sequencing (DRS), LC–MS/MS, and yeast genetics to profile T-loop modifications across all 42 budding yeast cytosolic tRNA isoacceptors. Our results established the most comprehensive list of the Ψ_55_–m^1^A_58_ T-loop modification circuit known from any organism. Moreover, we discovered a novel nutrient-dependent increase in m^1^A_58_ on some isoacceptors. We also used bioinformatic analysis to build a T-loop modification classifier permitting prediction of modifications from DRS data.

While our study has not entered the realm of single-molecule analysis of RNA modifications, it lays a foundation. To achieve single-molecule analysis, a combination of DRS and MS should be applied to a set of oligonucleotide standards containing specified combinations of modifications, such as the three involved in this study. MS-based analytical measurements of these RNAs, followed by sequencing, could be used to train DRS to detect a specific ensemble of modifications in *individual* tRNAs from cells, potentially quantitatively. Due to the complexity of developing these reagents—a multitude of combinations of modifications for even a single isoacceptor, each requiring cross-validation by MS—this work is beyond the scope of this study. Such reagents will be important, however, for advances toward quantitative, single-molecule analysis of RNA modifications by DRS.

Our study will draw obvious comparisons to a recent publication presenting ‘Nano-tRNAseq’ (6), due to similar methodology and results on yeast cytosolic tRNAs. The duplexed adaptor methodology presented in the current study was identical in design to our previous work on *E. coli* tRNAs (5), whereas the Nano-tRNAseq used a similar adaptor design but with an additional reverse transcriptase step to linearize the tRNA, which we did not perform. A major focus of the Lucas *et al.* paper was to improve throughput, which they achieved primarily by optimizing the data collection software settings. While both studies provided evidence for the ability of Nanopore sequencing to document previously identified T-loop modification circuits (19), our study addresses this topic more thoroughly.

The study herein examined base calling errors in the T-loop in WT and in genetic knockouts for the three enzymes responsible for T-loop modifications. We also employed an error model based on IVT controls for all 42 isoacceptors, to generate posterior probabilities at each nucleotide position. In contrast, the Lucas *et al.* study used base calling rates in WT versus *pus4*Δ, a genetic knockout of one of the three enzymes, to make its conclusions. While they reported Nanopore base miscall signal associated with the m^5^U_54_ modification, in contrast, we found that the base calling error due to m^5^U_54_ was close to the background Nanopore error, using the *trm2*Δ yeast strain. Moreover, our use of the *trm6*Δ strain, that removes m^1^A_58_, permitted clear delineation of the base calling error associated with this modification. This in turn provided evidence and prediction of the m^1^A_58_ modification in isoacceptors not yet documented in Modomics. As both our and the Lucas *et al.* studies demonstrated, total nucleoside mass spectrometry measurements can improve interpretation of Nanopore sequencing results. Our study extended mass spectrometry analysis to a bottom-up RNA-sequencing dataset, permitting direct validation of the position of modifications in specific isoacceptor T-loops that had been predicted by DRS.

Currently, *E. coli* and yeast are the only organisms for which the sequences, including many but not all modifications, of most of the tRNA isoacceptors are known, as referenced in the Modomics database. For other organisms, however, there remains a limited catalog of isoacceptor sequences and an even more limited catalog of their RNA modifications. Our study showcases the capability for DRS, particularly when supported with LC–MS/MS analysis and genetics, to measure the interdependence of multiple and closely spaced tRNA modifications at transcriptome scale.

We observed different patterns of the m^1^A_58_ modification by the Trm6 + Trm61 complex depending on how cells were grown. Under exponential growth conditions, we observed 24/42 isoacceptors having an m^1^A signal within positions 57–59, however this increased to 27/42 isoacceptors once cells reached saturation. Because studies may perform analysis of RNA modifications under different growth conditions, it is important to consider that reported differences in modifications can arise due to differences in how cells were grown. In Supplementary Table S9 we summarized comparisons of m^1^A_58_ annotations from three studies including ours, mim-tRNA-seq (4), and ARM-seq (48). In this study cells were harvested either at OD_600_ ∼0.8 or at a saturated phase (beyond OD_600_ 2.0). In the mim-tRNA-seq study, cells were reported harvested at OD_600_ ∼0.5. And in ARM-seq, cells were reported harvested between OD_600_ 1.0 and 2.0. It is possible that these differences in growth phase of the cells could lead to variability in the presence of this modification. Combining our DRS data from both growth conditions produced highest agreement across the three studies, in terms of predicting/reporting the presence of m^1^A_58_ in at least one of our growth conditions compared to the other two studies. Therefore differences between modification annotations from different studies may sometimes be reconciled through consideration of the growth requirements or environmental conditions. These data are reminiscent of many previous studies documenting how the level of certain tRNA chemical modifications can change during stress or altered growth states, across kingdoms of life (6,50,61,67,76–101). Similar considerations should also be made for multicellular organisms, in which it is expected that different tissues from the same organism might display different modification patterns even on the same tRNA isoacceptors.

Our multiclass model for predicting m^1^A_58_ in tRNA isoacceptors using base miscall information not only enabled our discovery that m^1^A_58_ is increased significantly on some isoacceptors in yeast cells undergoing nutrient starvation, but it promises utility beyond yeast. We note that human tRNA isoacceptors share significant identity in their T-loop sequences with those in yeast—approximately half of known human T-loop sequences share identical 7-mers to the yeast isoacceptors analyzed in this study. Therefore this type of classification model could permit analysis of T-loop modification dynamics or circuits in human tRNA. It is also possible that modification circuits that appear common in yeast T-loops may be conserved in other organisms. If so, it will be useful to have synthetic control tRNA molecules with different combinations of entailed modifications, for improved model training. And because it is possible that deletion of a modification catalyzing enzyme could enable other RNA modifying enzymes to ectopically modify the same position, or a neighboring position, LC–MS/MS will be essential for verification of modifications.

Forthcoming technological improvements will steadily address current limitations associated with tRNA analysis by Nanopore DRS. For example, tRNA sequence analyses are limited by baseline DRS base calling accuracy, and an ONT-sourced basecaller that is not specially trained on shorter RNA molecules, such as tRNAs. Median Nanopore DRS accuracy has improved from 86% in 2019 (102) to 97.3% in 2024 (unpublished data, M. Jain, Northeastern University). In the current study we relied on comparison of reference match probabilities between different strains or conditions, but the numerical variation in probabilities at specific sites across different isoacceptors do not represent a quantitative measurement of the abundance of a modification.

Ionic current analysis of tRNA molecules is essential for quantitative learning of signal differences between unmodified and modified nucleotides, and thus prediction of modification stoichiometry. Such analysis has already been done for some rRNA and mRNA modifications (31). Presently, most DRS-based modification analysis tools use either Nanopolish (102) or Tombo algorithms (103) to convert the raw ionic current data into mean current amplitude (pA), standard deviation (pA), and dwell time (s) with 5-mers in individual aligned reads. This approach has been applied for detecting m^6^A (104) (105,106), inosine (107,108), and pseudouridine (109). For tRNA, however, these approaches present challenges because the signal generated from the complete translocation of a tRNA through a flow cell pore is brief (typically <1 s), relative to the Nanopore sequencing adapter that is composed of DNA bases that take longer to translocate through the pore (typically 2–4 s). Since ionic current analysis tools like Nanopolish require RNA-based ionic current signal periods that exceed the length from the DNA-based adaptors (typically >4 s), the adapted tRNA molecules as presented above require further modifications in order for these programs to convert their raw ionic current signal into base calls.

To address this need, we extended the length of the splint adapters using RNA nucleotides to thereby increase the RNA-associated translocation time. This increased ionic current signal sufficiently to permit Nanopolish kmer-level alignments for yeast tRNAs. For the 5′-tRNA splint adapter we increased the number of ribonucleotides from 18 to 120, and for the 3′-tRNA splint adapter we increased from 6 to 46 ribonucleotides (Supplemental Figure 8A). We then sequenced wild-type yeast tRNA using these longer splint adapters. An example of the tRNA^Pro(UGG)^ isoacceptor with long adapters is shown in Supplemental Figure 8B. When accounting for all 42 isoacceptors from this experiment, 10 901 single tRNA reads out of 47 612 total aligned reads, or 23%, could be analyzed by Nanopolish. In contrast, with the shorter adapters for wild-type tRNA samples, *zero* reads were amenable to Nanopolish analysis. These longer adapters on tRNAs demonstrate that it is possible to perform ionic current-based modification analysis for shorter RNA molecules. For future studies on tRNAs and other shorter RNA molecules, we therefore recommend using longer adapter sequences to enable ionic current analysis of tRNAs using existing software like Nanopolish. This will enable robust and potentially quantitative analysis of tRNA modifications using DRS.

Measuring modifications in other segments of tRNAs by DRS, particularly the anticodon loop where they most directly influence protein synthesis, will yield additional insights into the coordination of tRNA chemical modifications across environments and organisms. The variety and combinatorial complexity of RNA modifications in these regions far surpass those in the T-loop. Therefore, at minimum, IVT controls, and biological controls that remove an RNA modification of interest are critical for interpreting how that modification influences the Nanopore signal. We propose that the combination of these controls, along with LC-MS/MS measurements, represent the ‘best practices’ approach for analyzing RNA modifications using Nanopore DRS.

Finally, in the future Nanopore DRS can also be used to study questions regarding tRNA biogenesis. Mature tRNAs result from a well-stereotyped process of the synthesis of a pre-tRNA transcript that contains a 5′ leader sequence and 3′ trailer sequence that are subsequently cleaved, the non-templated addition of the 3′-CCA end, as well as the splicing of introns in some tRNAs. The timing and coordination of these different steps, in conjunction with the catalysis of various modifications, and in response to cellular stress or different growth conditions, could be studied in a systematic way using Nanopore DRS. Efforts required include establishing an adaptor/sequencing design that captures the various precursors, as the adaptors used in the current study are only designed to capture mature tRNA molecules with canonical 5′ and 3′ ends. While we have already mapped intron-containing reads in a minority of molecules for certain isoacceptors (data not shown), the use of certain mutants will promote enrichment of these precursor intron-containing molecules, together with deeper sequencing of wild-type strains. Together these approaches could significantly enhance the systems-wide view of tRNA maturation and chemical modification in ‘normal’ and stress conditions, as well as in human disease.

## Supplementary Material

gkae796_Supplemental_Files

## Data Availability

The sequencing data described in this manuscript are publicly accessible at the European Nucleotide Archive https://www.ebi.ac.uk/ena. The study accession number is PRJEB68201.
