# Supplementary material for: Combining Nanopore direct RNA sequencing with genetics and mass spectrometry for analysis of T-loop base modifications across 42 yeast tRNA isoacceptors
Source: Nucleic Acids Res. 2024 Sep 28;52(19):12074–92. doi: 10.1093/nar/gkae796 (PMC11514469; doi:10.1093/nar/gkae796)
Supplement: gkae796_Supplemental_Files [file gkae796_supplemental_files.zip › gkae796_Supplementary_Data.pdf]

Table S1. Yeast strains used in this study.

Strain	Phenotype	Genotype	Source
YDG1	Wild-type	MATa, *his3*Δ1, *leu*2Δ0, *met15*Δ0, *ura3*Δ0	This study
YDG127	Lacks Ψ_{55}	MATa, *PUS4*::KanMX, *his3*Δ1, *leu2*Δ0, *met15*Δ0, *ura3*Δ0	Yeast Knockout Library<br><br>(1)
YDG221	Lacks Ψ_{55}	MATα, *PUS4*::KanMX, *his3*Δ1, *leu2*Δ0, *lys2*Δ0, *ura3*Δ0	Yeast Knockout Library<br><br>(1)
YDG630	Catalytically dead Pus4. Lacks Ψ_{55}	MATa, *PUS4*-R286K, *his3*Δ1, *leu2*Δ0, *met15*Δ0, *ura3*Δ0	This study
YDG963	Lacks m^5U_{54}	MATa, *TRM2*::KanMX, *his3*Δ1, *leu2*Δ0, *met15*Δ0, *ura3*Δ0	Yeast Knockout Library<br><br>(1)
YDG973	Lacks Ψ_{55} and m^5U_{54}	*PUS4*::KanMX, *TRM2*::KanMX, *his3*Δ1, *leu2*Δ0, *ura3*Δ0	This study
H2846 (YJA146)	Lacks m^1A_{58}	MATα, *TRM6*::HisG, *ura3-52*, *trp2*, *leu2*Δ, *his3*Δ, *PEP*::HIS3, *prb1*Δ, *can1*, GAL+ (plasmid h.c. IMT4, LEU2)	(2)

H2847	Lacks m^1A_{58}	*TRM61*::URA3, *ura3-52*, *leu2-3*, *leu2-112*, *trp1-289*, *his3-111*, *can1-100*, ADE+ (plasmid h.c. IMT4, LEU2)	(2)
YDG989	C-terminal 3xHA tag on Pus4	MATa, *his3*Δ1, *leu2*Δ0, *met15*Δ0, *ura3*Δ0, *PUS4*-3xHa	This study
YDG1033	C-terminal 3xHA tag on Pus4-R286K	MATa, *his3*Δ1, *leu2*Δ0, *met15*Δ0, *ura3*Δ0, *PUS4*-3xHA-R286K	This study

Table S2. The RNA and DNA oligonucleotides for the splint adapters and IVT constructs, and the oligos and gBlocks used for construction of the CRISPR plasmids and the repair templates.
See Supplemental_Tables_S2_S5_S7_S8_S10_S12.xlsx

Table S3. Global and isoacceptor-specific profiles. This sheet provides information about how training and testing data were labeled, the depth of data used, and the accuracy of the models. It should be noted that not all profiles correspond to the presence or absence of a single modification due to the effect of modification circuits (e.g. *pus4*Δ results in the loss of both Ψ_{55} and m^1A_{58}). These data are from exponentially growing cells.
See Supplemental_Table_S3_Sequence_Level_Classification_Statistics.xlsx

Table S4. Sequence and alignment statistics for tRNA purified from *S. cerevisiae* strains (wild-type, *pus4*Δ, *trm6*Δ, *trm61*Δ, *trm2*Δ, *pus4*Δ*trm2*Δ, and *PUS4* R286K) and for *in vitro* transcribed tRNA. "N" represents the number of biological replicates. Alignments were generated using a reference that contained the adapter sequences and were filtered at MAPQ>0.

**Sample**	**Per flow cell**			
	**Total reads**	**Aligned tRNA reads**	**Full-length aligned tRNA reads**	**Full-length tRNA reads as a percent of aligned tRNA reads**
Wild-type N = 4	67 200 – 95 451	27 882 – 37 800	24 261 – 32 938	87.23 – 91.45%
*pus4*Δ N = 3	99 165 – 113 095	51 635 – 59 884	28 485 – 48 205	89.41 – 91.45%
*trm6*Δ N = 3	23 452 – 138 054	6194 – 42 767	5354 – 36 588	85.75 – 86.44%
*trm61*Δ N = 1	124 939	42 607	35 157	82.51%
*trm2*Δ N = 3	22 580 – 45 849	10 834 – 17 960	9520 – 36 175	87.87 – 89.09%
*pus4*Δ *trm2*Δ N = 3	25 908 – 383 570	13 374 – 200 097	11 643 – 176 647	87.06 – 88.62%
PUS4 R286K N = 3	41 184 – 128 053	14 594 – 69 698	12 346 – 59 214	84.93 – 86.42%
IVT N = 1	603 915	515 091	466 251	90.52%

Table S5. Reference sequences for 42 yeast cytosolic tRNA isoacceptors from strain BY4741. See Supplemental_Tables_S2_S5_S7_S8_S10_S12.xlsx

Table S6. Isoacceptor aligned read ranges across four wild-type *S. cerevisiae* replicates.

Isoacceptor	Aligned reads (MAPQ>0)	Isoacceptor	Aligned reads (MAPQ>0)
Ala (AGC)	655–2803	Leu (UAA)	73–1150
Ala (UGC)	1060–1335	Leu (UAG)	129–726
Arg (ACG)	1319–1879	Lys (CUU)	957–4878
Arg (CCG)	182–532	Lys (UUU)	248–1517
Arg (CCU)	187–551	iMet (CAU)	13–580
Arg (UCU)	823–1714	Met (CAU)	324–444
Asn (GUU)	1034–1544	Phe (GAA)	725–1322
Asp (GUC)	3191–4403	Pro (AGG)	100–799
Cys (GCA)	333–1145	Pro (UGG)	177–320
Gln (CUG)	222–555	Ser (AGA)	2357–9565
Gln (UUG)	572–1583	Ser (CGA)	7–180
Glu (CUC)	194–229	Ser (GCU)	24–1028
Glu (UUC)	1272–1501	Ser (UGA)	3–106
Gly (CCC)	203–467	Thr (AGU)	1059–2413
Gly (GCC)	2195–10 978	Thr (CGU)	78–171
Gly (UCC)	275–504	Thr (UGU)	63–198
His (GUG)	378–3887	Trp (CCA)	241–734
Ile (AAU)	978–1347	Tyr (GUA)	440–1717
Ile (UAU)	119–280	Val (AAC)	1390–1811
Leu (CAA)	1170–12 237	Val (CAC)	263–452
Leu (GAG)	120–179	Val (UAC)	356–1072

Table S7. Raw aligned reads for each replicate from wild-type, *pus4*Δ, *trm6*Δ, *trm2*Δ, *pus4*Δ*trm2*Δ, and *trm61*Δ. See Supplemental_Tables_S2_S5_S7_S8_S10_S12.xlsx

Table S8. The T-loop sequences from 42 cytosolic *S. cerevisiae* isoacceptors. See Supplemental_Tables_S2_S5_S7_S8_S10_S12.xlsx

Table S9. Measurements or predictions of the presence or absence of m^1A_{58} across 42 cytosolic tRNA isoacceptors in wild-type yeast, from several experimental approaches. Blue circles indicate/predict presence. Magenta circles indicate/predict absence. No circle represents an inconclusive result. Modomics annotations are from reference (3). DRS predictions are blue/present if the reference nucleotide posterior probabilities at positions 57, 58, or 59 fell below the threshold cutoff of 0.7. While $tRNA^{Arg(CCU)}$ fell below this threshold in both exponentially growing and saturated cells, we do not indicate modification since the same change was observed in *trm6*Δ grown in each condition (**Figure 3c** for exponential growth data). For DRS, boxes containing one solid circle indicate that those predictions were observed in *both* exponentially growing *and* saturated cells. Boxes with two circles indicate predictions from exponentially growing (left circle) and saturated cells (right circle). Evidence for m^1A_{58} through LC-MS/MS was taken from sequence informative fragmentation ions (4). mim-tRNAseq m^1A_{58} predictions were considered positive for isoacceptors with misincorporation rates >10% and nucleotide coverage >2000 reads at position 58 (5). ARM-seq m^1A_{58} predictions were considered positive for isoacceptors with a two-fold or greater increase in the ratio of read counts from AlkB-treated versus untreated RNA and a p<0.01 (6).

tRNA	Modomics	DRS	LC-MS/MS	mim-seq	ARM-seq
AlaAGC	magenta	magenta	magenta	magenta	magenta
AlaUGC		magenta		magenta	magenta
ArgACG	blue	blue	blue	blue	blue
ArgCCG		magenta		magenta	magenta
ArgCCU		magenta		magenta	magenta
ArgUCU	blue	blue	blue	blue	blue
AsnGUU	blue	blue	blue	blue	blue
AspGUC	magenta	magenta	magenta	magenta	magenta
CysGCA	blue	blue		blue	blue
GlnCUG		blue		blue	blue
GlnUUG		blue		blue	blue
GluCUC		magenta		magenta	magenta
GluUUC	magenta	magenta	magenta	magenta	magenta
GlyCCC		magenta		magenta	magenta
GlyGCC	magenta	magenta		magenta	magenta
GlyUCC	magenta	magenta		magenta	magenta
HisGUG	magenta	magenta	magenta	magenta	magenta
IleAAU	blue	blue	blue	blue	blue
IleUAU	blue	blue	blue	blue	blue
LeuCAA	magenta	magenta	magenta	magenta	magenta
LeuGAG		blue	blue	blue	blue
LeuUAA	blue	magenta, blue		blue	magenta
LeuUAG	blue	blue	blue	blue	blue
LysCUU	blue	magenta, blue		blue	magenta
LysUUU	blue	blue		blue	blue
MetCAU	blue	blue		blue	blue
iMetCAU	blue	blue	blue	blue	blue
PheGAA	blue	blue	blue	blue	blue
ProAGG		magenta		magenta	magenta
ProUGG	blue	blue	blue	blue	blue
SerAGA	magenta	magenta	magenta	magenta	magenta
SerCGA	magenta	blue		blue	blue
SerGCU	blue	blue	blue	blue	blue
SerUGA	magenta	magenta		magenta	magenta
ThrAGU	blue	blue	blue	blue	blue
ThrCGU		blue	blue	blue	blue
ThrUGU		blue		blue	blue
TrpCCA	blue	blue	blue	blue	blue
TyrGUA	blue	blue		blue	blue
ValAAC	blue	blue	blue	blue	blue
ValCAC	blue	magenta, blue	blue	blue	magenta
ValUAC	blue	blue		blue	blue

Table S10. The posterior probabilities of an alternative nucleotide in the 3-mer window where the m^1A_{58} signal occurs (positions 57–59), and at the single position 54 where m^5U occurs. Each probability is derived from 120 aligned reads. If the probability was ≥0.3, it passed the threshold that we considered to be a bonafide miscall.
See Table S10 on Supplemental_Tables_S2_S5_S7_S8_S10_S12.xlsx

Table S11. Classes of tRNAs as defined by the influence of Ψ_{55} on the catalysis of m^1A_{58} *in exponentially growing cells*. Class I tRNAs do not have Ψ_{55}, but do have m^1A_{58}. Class II tRNAs have Ψ_{55}, but do not have m^1A_{58}. Class III tRNAs have both Ψ_{55} and m^1A_{58}, and the presence of Ψ_{55} promotes m^1A_{58} catalysis. Class IV tRNAs have both Ψ_{55} and m^1A_{58}, but the loss of Ψ_{55} does not substantially reduce m^1A_{58} modification.

	**Class I**	**Class II**	**Class III**	**Class IV**
**tRNA isoacceptor**	iMet (CAU)	Ala (AGC)<br>Ala (UGC)<br>Arg (CCG)<br>Arg (CCU)<br>Asp (GUC)<br>Glu (CUC)<br>Glu (UUC)<br>Gly (CCC)<br>Gly (GCC)<br>Gly (UCC)<br>His (GUG)<br>Leu (CAA)<br>Leu (UAA)<br>Lys (CUU)<br>Pro (AGG)<br>Ser (AGA)<br>Ser (UGA)<br>Val (CAC)	Arg (UCU)<br>Asn (GUU)<br>Cys (GCA)<br>Gln (CUG)<br>Gln (UUG)<br>Ile (AAU)<br>Leu (GAG)<br>Leu (UAG)<br>Lys (UUU)<br>Met (CAU)<br>Phe (GAA)<br>Pro (UGG)<br>Ser (CGA)<br>Ser (GCU)<br>Thr (CGU)<br>Thr (UGU)<br>Trp (CCA)<br>Tyr (GUA)<br>Val (AAC)	Arg (ACG)<br>Ile (UAU)<br>Thr (AGU)<br>Val (UAC)

Table S12. Mass spectrometry ribonucleoside modification profiling abundances. Total tRNA was isolated from wild-type, *pus4*Δ, *trm6*Δ, *trm2*Δ, and *pus4*Δ*trm2*Δ strains. The average percentage of a modified nucleoside was taken from three biological replicates. Standard deviations were calculated for the abundance of every modification for each strain. P-values taken from a t-test are displayed showing the statistical significance of the difference between modification abundance in comparison to wild-type. The percentage change represents the change in the level of a modification from wild-type to indicated mutant strain.
See Supplemental_Tables_S2_S5_S7_S8_S10_S12.xlsx

Figure S1

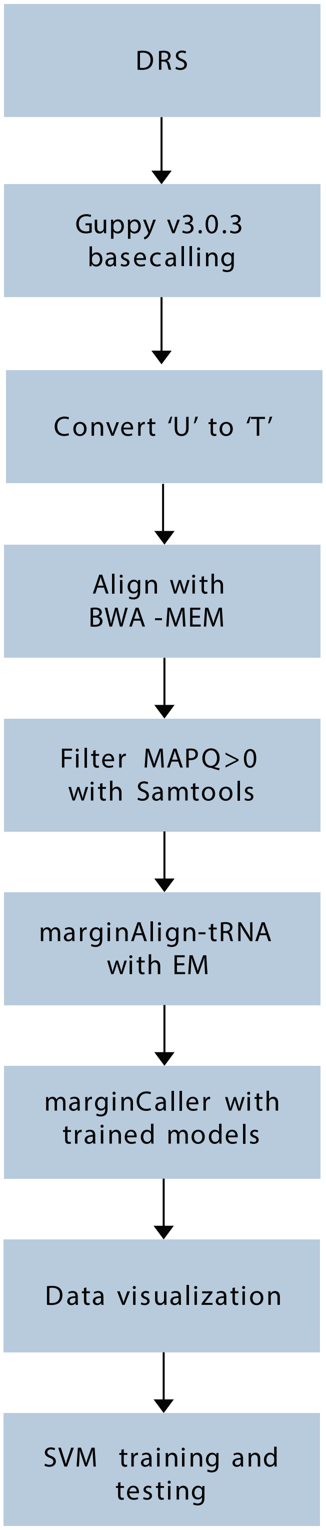

Supplementary Figure 1. A general overview of the computational analysis pipeline. Direct RNA sequencing (DRS) data were collected and base called with Guppy v3.0.3. All 'U' calls were converted to 'T' before alignment to the reference with BWA-MEM, and then alignments were filtered for MAPQ>0. Alignment and error models were generated using marginAlign with expectation maximization (EM) training activated. These alignments and accompanying models were then run through marginCaller to generate posterior probabilities (mismatch probabilities) for every position. Posterior probabilities were visualized with heatmaps and used to determine labeling of samples in Support Vector Machine (SVM) training and testing. See Methods for software setting details.

Figure S2

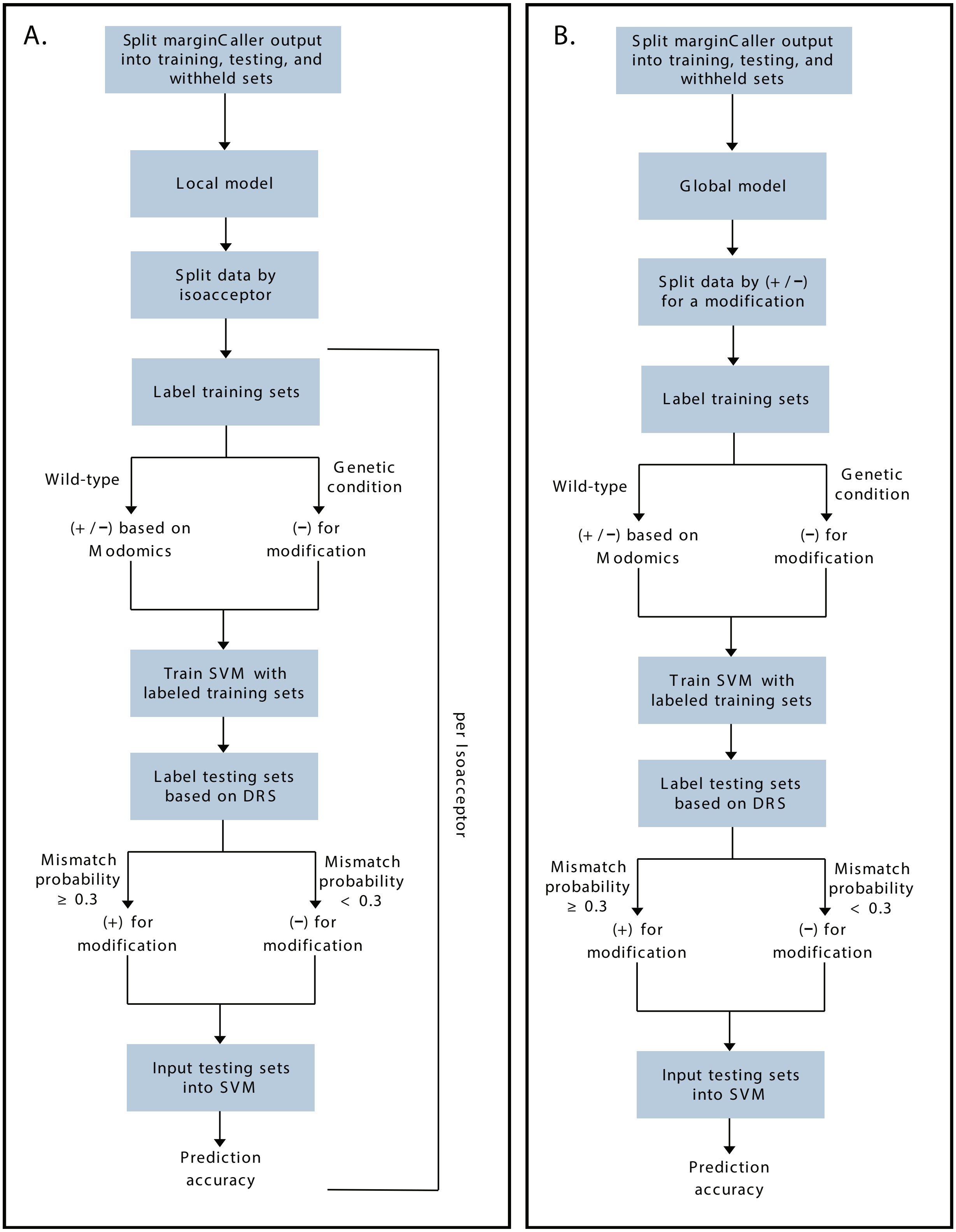

Supplementary Figure 2. An overview of Support Vector Machine training and testing. Flow charts of the Support Vector Machine (SVM) training and testing of the local and global modification profile prediction models. **a)** In the local model (Isoacceptor model), data was split and processed per isoacceptor and labeled by presence or absence of T-loop modification(s). The training sets were labeled based on Modomics and the testing sets were labeled based on a posterior probability (Mismatch probability) cutoff of ≥ 0.3. **b)** In the global model data was split into sets of isoacceptors based on presence or absence of particular T-loop modifications, using Modomics as ground truth. The training sets were labeled based on Modomics and the testing sets were labeled based on a posterior probability cutoff of ≥ 0.3.

Figure S3

```
   AA-stem  D-stem             AC-stem            V-loop helix   T-stem
   (((((((..((((..........)))).(((((.......))))).................(((((.......))))))))))))....
   12345678901234567890ab1234567890123456789012345VVVVVVVVVVVV6789012345678901234567890123456
   0        1         2           3         4     ABCDEFGHIJKL    5         6         7
A  GGGCGUGUGGCGUAGUCGGU**AGCGCGCUCCCUUAGCAUGGGAGAG************GUCUCCGGUUCGAUUCCGGACUCGUCCACCA
A  GGGCACAUGGCGCAGUUGGU**AGCGCGCUUCCCUUGCAAGGAAGAG************GUCAUCGGUUCGAUUCCGGUUGCGUCCACCA
R  UUCCUCGUGGCCCAAU*GGUC*ACGGCGUCUGGCUACGAACCAGAAG************AUUCCAGGUUCAAGUCCUGGCGGGGAAGCCA
R  GCUCCUCUAGUGCAAU*GGUU*AGCAUGCAUUCUUCCGGUGGCUGUG************A*UCCGGGUUCGAGUCCCGGGAGGAGCUCCA
R  GUUCCGUUGGCGUAAU*GGU**AACGCGUCUCCCUCCUAAGGAGAAG************ACUGCGGGUUCGAGUCCCGUACGGAACGCCA
R  GCUCGCGUGGCGUAAU*GGC**AACGCGUCUGACUUCUAAUCAGAAG************AUUAUGGGUUCGACCCCCAUCGUGAGUGCCA
N  GACUCCAUGGCCAAGUUGGUU*AAGGCGUGCGACUGUUAAUCGCAAG************AUCGUGAGUUCAACCCUCACUGGGGUCGCCA
D  UCCGUGAUAGUUUAAU*GGUC*AGAAUGGGCGCUUGUCGCGUGCCAG************A*UCGGGGUUCAAUUCCCCGUCGCGGAGCCA
C  GCUCGUAUGGCGCAGU*GGU**AGCGCAGCAGAUUGCAAAUCUGUUG************GUCCUUAGUUCGAUCCUGAGUGCGAGCUCCA
Q  GGUCCUAUAGUGUAGU*GGUU*AUCACUUUCGGUUCUGAUCCGAACA************A*CCCCAGUUCGAAUCCGGGUGGGACCUCCA
Q  GGUCCUAUAGUGUAGU*GGUU*AUCACUUUCGGUUUUGAUCCGGACA************A*CCCCGGUUCGAAUCCGGGUAGGACCUCCA
E  UCCGAUGUAGUGUAAC*GGCU*AUCACAUCACGUUCUCACCGUGGAG************A*CGGGGUUCGACUCCCCGCUUCGGAGCCA
E  UCCGAUAUAGUGUAAC*GGCU*AUCACAUCACGCUUUCACCGUGGAG************A*CGGGGUUCGACUCCCCGUAUCGGAGCCA
G  GCGCAAGUGGUUCAGU*GGUU*AGAAUUUAUGCUUCCCAAGCAUGAG************G*CCCGGGUUCGAUUCCCGGCUUGCGCACCA
G  GCGCAAGUGGUUUAGU*GGU**AAAAUCCAACGUUGCCAUCGUUGGG************C*CCCCGGUUCGAUUCCGGGCUUGCGCACCA
G  GGGCGGUUAGUGUAGU*GGUU*AUCAUCCCACCCUUCCAAGGUGGGG************A*CACGGGUUCGAUUCUCGUACCGCUCACCA
H GGCCAUCUUAGUAUAGU*GGUU*AGUACACAUCGUUGUGGCCGAUGA*************AACCCUGGUUCGAUUCUAGGAGAUGGCACCA
I  GGUCUCUUGGCCCAGUUGGUU*AAGGCACCGUGCUAAUAACGCGGGG************AUCAGCGGUUCGAUCCCGCUAGAGACCACCA
I  GCUCGUGUAGCUCAGU*GGUU*AGAGCUUCGUGCUUAUAACGCGACC************GUCGUGGGUUCAAACCCCACCUCGAGCACCA
L  GGUUGUUUGGCCGAGC*GGUCUAAGGCGCCUGAUUCAAGCUCAGGU**AUCGUAAGAU*G*CAAGAGUUCGAAUCUCUUAGCAACCACCA
L  GGUACUCUGGCCGAGU*GGUCUAAGGCGUCAGGUCGAGGUCCUGAU**CUCUUCGGAG*G*CGCGGGUUCAAACCCCGCGGGUAUCACCA
L  GGAGGGUUGGCCGAGU*GGUCUAAGGCGGCAGACUUAAGAUCUGUU*GGACGGUUGUCCG*CGCGAGUUCGAACCUCGCAUCCUUCACCA
L  GGGAGUUUGGCCGAGU*GGUUUAAGGCGUCAGAUUUAGGCUCUGAU**AUCUUCGGAU*G*CAAGGGUUCGAAUCCCUUAGCUCUCACCA
K  GCCUUGUUGGCGCAAUCGGU**AGCGCGUAUGACUCUUAAUCAUAAG************GUUAGGGGUUCGAGCCCCCUACAGGGCUCCA
K  UCCUUGUUAGCUCAGUUGGU**AGAGCGUUCGGCUUUUAACCGAAAU************GUCAGGGGUUCGAGCCCCCUAUGAGGAGCCA
M  AGCGCCGUGGCGCAGU*GGA**AGCGCGCAGGGCUCAUAACCCUGAU************GUCCUCGGAUCGAAACCGAGCGGCGCUACCA
M  GCUUCAGUAGCUCAGUAGGA**AGAGCGUCAGUCUCAUAAUCUGAAG************GUCGAGAGUUCGAACCUCCCCUGGAGCACCA
F  GCGGAUUUAGCUCAGUUGGG**AGAGCGCCAGACUGAAGAUCUGGAG************GUCCUGUGUUCGAUCCACAGAAUUCGCACCA
P  GGGCGUGUGGUCUAGA*GGU**AUGAUUCUCGCUUAGGGUGCGGGAG************GUCCCGGGUUCGAGUCCCGGCUCGCCCCCCA
P  GGGCGUGUGGUCUAGU*GGU**AUGAUUCUCGCUUUGGGUGCGAGAG************GCCCUGGGUUCAAUUCCCAGCUCGCCCCCCA
S  GGCAACUUGGCCGAGU*GGUU*AAGGCGAAAGAUUAGAAAUCUUUU*GGGCUUUGCCC*G*CGCAGGUUCGAGUCCUGCAGUUGUCGCCA
S  GGCACUAUGGCCGAGU*GGUU*AAGGCGAGAGACUCGAAAUCUCUU*GGGCUCUGCCC*G*CGCUGGUUCAAAUCCUGCUGGUGUCGCCA
S  GUCCCAGUGGCCGAGU*GGUU*AAGGCGAUGCCCUGCUAAGGCAUU*GGGUUUUACCU*G*CGCAGGUUCGAAUCCUGUCUGUGACGCCA
S  GGCACUAUGGCCGAGU*GGUU*AAGGCGACAGACUUGAAAUCUGUU*GGGCUCUGCCC*G*CGCUGGUUCAAAUCCUGCUGGUGUCGCCA
T  GCUUCUAUGGCCAAGUUGGU**AAGGCGCCACACUAGUAAUGUGGAG************AUCAUCGGUUCAAAUCCGAUUGGAAGCACCA
T  GCCCUUUUGGCCAAGU*GGU**AAGGCAUCGCACUCGUAAUGCGGGG************AUCGUGGGUUCAAUUCCCACAGAGGGCACCA
T  GCCUCCUUAGCUUAGU*GGU**AGAGCGUUGCACUUGUAAUGCAAAG************GUCGUUAGUUCAAUUCUGACAGGUGGCACCA
W  GAAGCGGUGGCUCAAU*GGU**AGAGCUUUCGACUCCAAAUCGAAGG************GUUGCAGGUUCAAUUCCUGUCCGUUUCACCA
Y  CUCUCGGUAGCCAAGUUGGUUUAAGGCGCAAGACUGUAAAUCUUGAG************AUCGGGCGUUCGACUCGCCCCCGGGAGACCA
V  GGUUUCGUGGUCUAGUCGGUU*AUGGCAUCUGCUUAACACGCAGAAC************GUCCCCAGUUCGAUCCUGGGCGAAAUCACCA
V  GUUCCAAUAGUGUAGC*GGCU*AUCACGUUGCCUUCACACGGCAAAG************GUCCCGAGUUCGAUCCUCGGUUGGAACACCA
V  GGUCCAAUGGUCCAGU*GGUUCAAGACGUCGCCUUUACACGGCGAAG************AUCCCGAGUUCGAACCUCGGUUGGAUCACCA
   12345678901234567890ab1234567890123456789012345VVVVVVVVVVVV6789012345678901234567890123456
   0        1         2           3         4     ABCDEFGHIJKL    5         6         7
```

Supplementary Figure 3. Structured sequence alignment of 42 cytosolic tRNA isoacceptors from *S. cerevisiae*. Each base position of the alignment matches a box in the heatmap presented in **Figure 2**. Asterisks indicate gaps. One letter amino acid code at left. Left and right parentheses and colored regions show predicted helices/stems (labeled at top). Numbers (top line) indicate each base position in a repeating series of ten, or in bottom line every tenth position. Variable loop is up to 12 base positions in length, and alignment of these bases, as well as neighboring positions 44, 45, 46 and 47, in the Leucine and Serine isoacceptors, were anchored via predicted helices/stems in the loop. G•U wobbles are included in predicted stem regions and underlined, while nucleotides ~~crossed out~~ in predicted stem regions are not predicted to form traditional Watson/Crick/Franklin base pairs if unmodified.

Figure S4

a

Ψ55-synthase catalytic residue

S. cerevisiae	Pus4	SSGTYIRSLVSDIG	R286
E. coli	TruB	SKGTYIRTIIDDLG	R181
H. sapiens	TRUB1	GGGFYIRSLVSDIG	R256

b

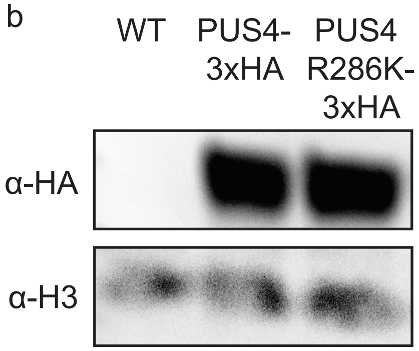

Supplementary Figure 4. Pus4 catalytic mutant. a) Protein sequence alignment of Pus4 and its *E. coli* and human homologs, showing the conserved catalytic arginine that was mutated to a lysine in the Pus4-R286K catalytic mutant. **b)** Western blot showing expression of HA-tagged Pus4-R286K catalytic mutant is comparable to HA-tagged wild-type Pus4. Histone H3 protein is probed as a loading control.

Figure S5

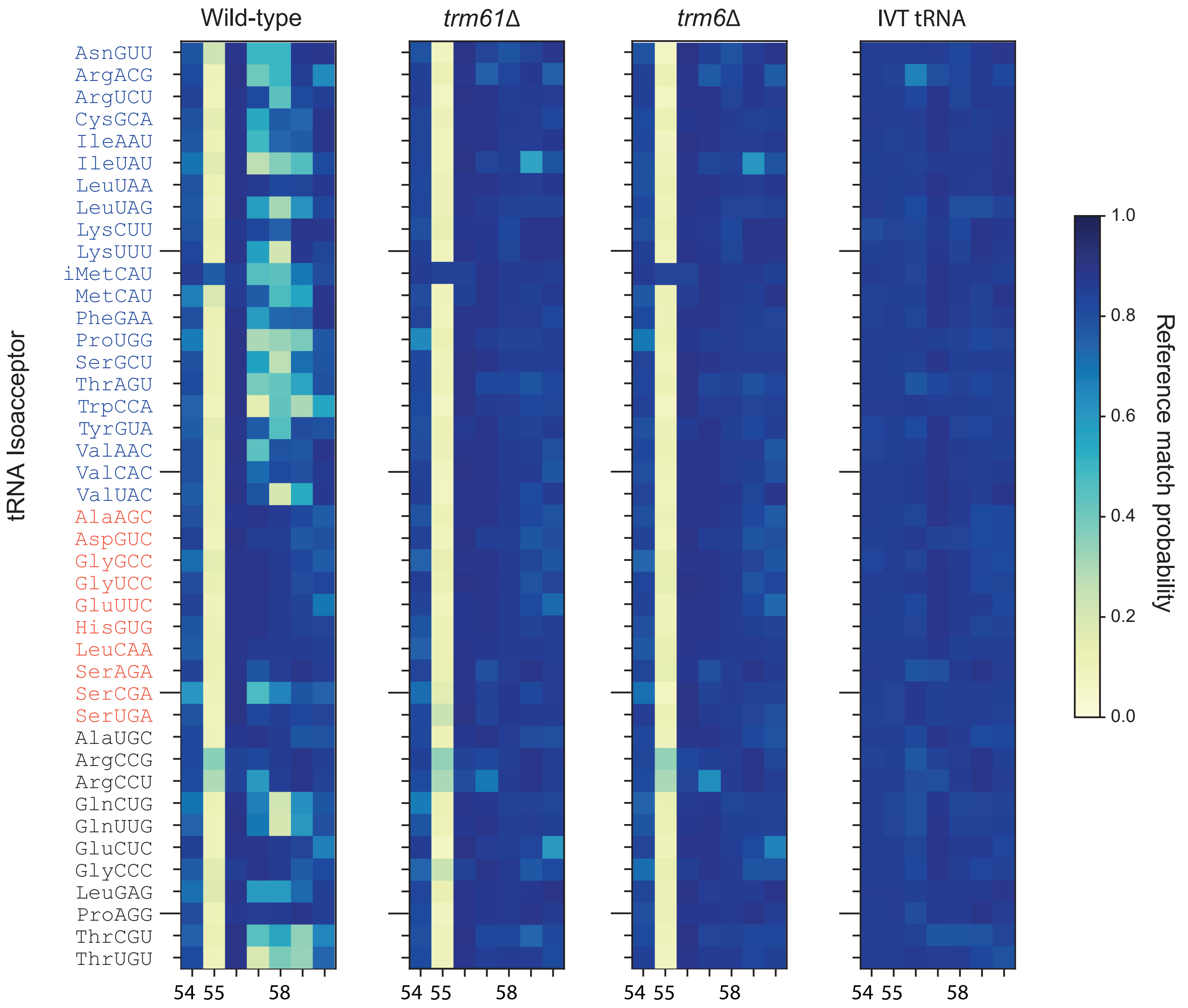

Supplementary Figure 5. Reference match probability map for *trm61*Δ match results obtained for *trm6*Δ. Reference match probability heat maps of aligned T-loop sequences across 42 tRNA isoacceptors, in wild-type, *trm61*Δ, *trm6*Δ, and *in vitro* transcribed (IVT). Note that wild-type, *trm6*Δ and IVT are reprinted from **Figure 3c**. Otherwise as described in **Figure 3c**.

Figure S6

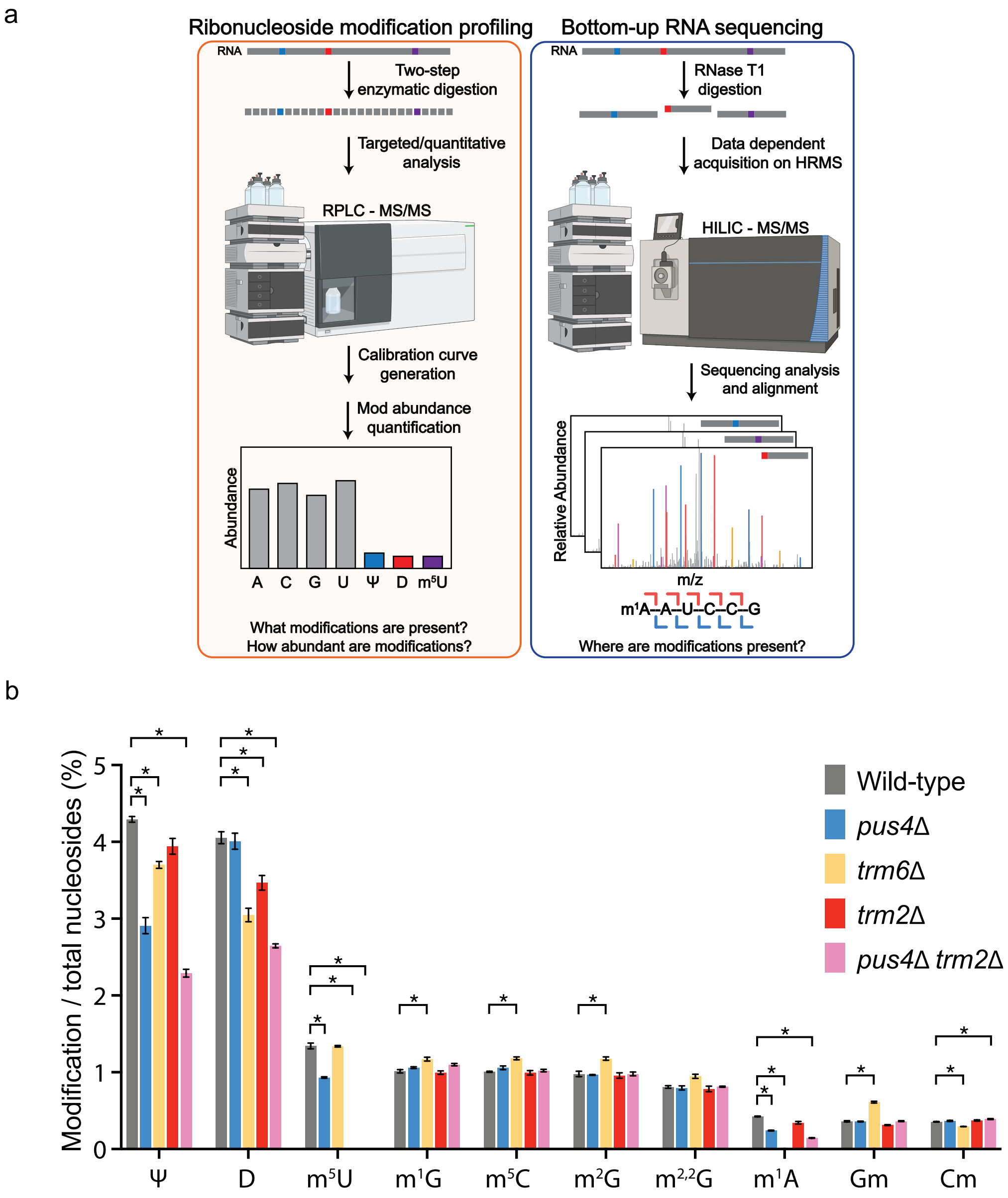

Supplementary Figure 6. Two different mass spectrometry techniques allow for the bulk quantification of each modification in a pool of total tRNA, or the specific location of RNA modifications. a) (left) Ribonucleoside modification profiling is performed from a two-step enzymatic digestion that degrades tRNA into monoribonucleosides. Reversed phase chromatography separates the ribonucleosides allowing for multiple reaction monitoring on a triple quadrupole mass spectrometer. This analysis allows for the quantification of individual RNA modifications from a pool of total tRNA. (right) Bottom-up RNA sequencing is performed by digestion of RNA usingRNase T1. Individual tRNA sequences can then be mapped using hydrophilic interaction liquid chromatography coupled to tandem mass spectrometry. Sequence informative fragmentation ions are detected in MS/MS spectra allowing for the specific location of RNA modifications to be determined. **b)** Each bar depicts the average percentage of the top 10 most abundant modified nucleosides (in wild-type), relative to total nucleosides, from three biological replicates. Total tRNA was taken from wild-type (grey), *pus4*Δ (blue), *trm6*Δ (yellow), *trm2*Δ (red), and *pus4*Δ,*trm2*Δ (pink). The error bars show the standard deviation, and significant changes ($p < 0.01$) are noted with an asterisk. In the *trm6*Δ strain, the relative abundance of tRNA$^{Leu(CAA)}$ changed from 13% to 52%. This change can help explain the increases observed in m^{1}G, m^{5}C, m^{2}G, m^{2,2}G, and Gm in the *trm6*Δ strain as tRNA$^{Leu(CAA)}$ contains all of these modifications.

Figure S7

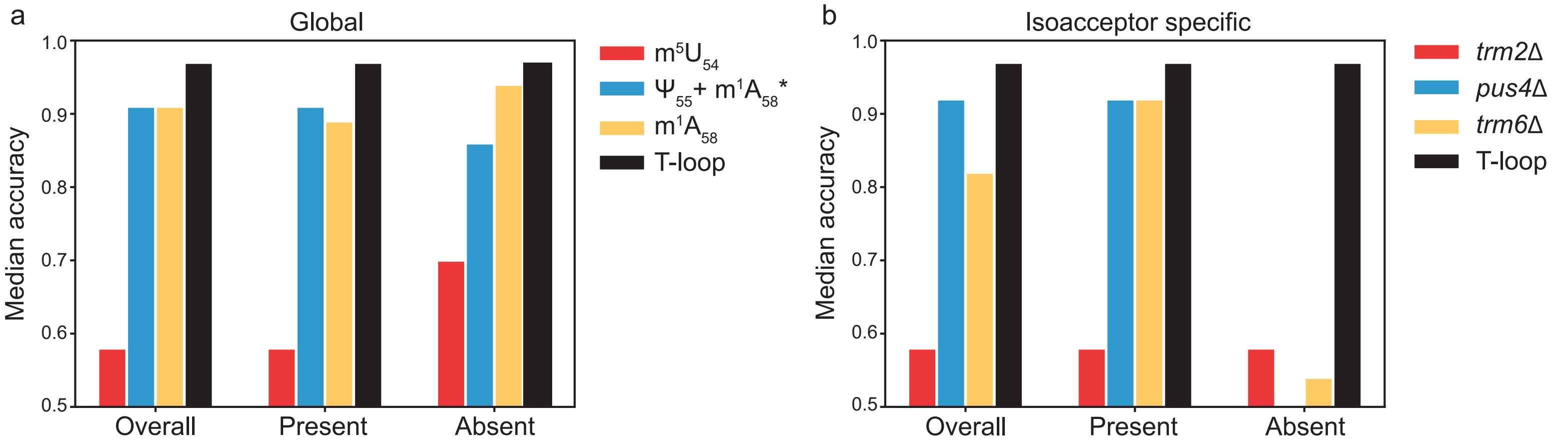

Supplementary Figure 7. m^5U_{54},Ψ_{55}, m^1A_{58} and T-loop modification associated miscall profile classification accuracies. Median accuracies indicate how accurately single molecule isoacceptor reads were classified as being positive or negative for the presence of m^5U_{54}, Ψ_{55} combined with m^1A_{58} (since these two are often coupled), m^1A_{58}, or all three possible modifications in the T-loop. **a)** Global profiles were generated with training data from isoacceptors that are known to have, or not have, m^5U_{54}, Ψ_{55}, m^1A_{58}, or all T-loop modifications in wild-type based on Modomics annotations, in comparison to corresponding knockout strains or unmodified samples (*trm2*Δ, *trm6*Δ, and IVT). For the Ψ_{55}+ m^1A_{58}* label, only isoacceptors annotated to have m^1A_{58} were used to train as "Present", and those annotated to *not* have it were used to train as "Absent". **b)** Isoacceptor-specific profiles were made similarly to global, but using isoacceptor-specific subsets. Training was done independently for each isoacceptor, with the assumption that the knockout strain would remove the corresponding modification, and IVT would have none. The median accuracy for the isoacceptor specific *pus4*Δ "Absent" profile is 0.50 because tRNA iMet lacks Ψ_{55} (its wild-type and pus4Δ profiles are the same). See **Supplementary Table 3** for additional details.

Figure S8

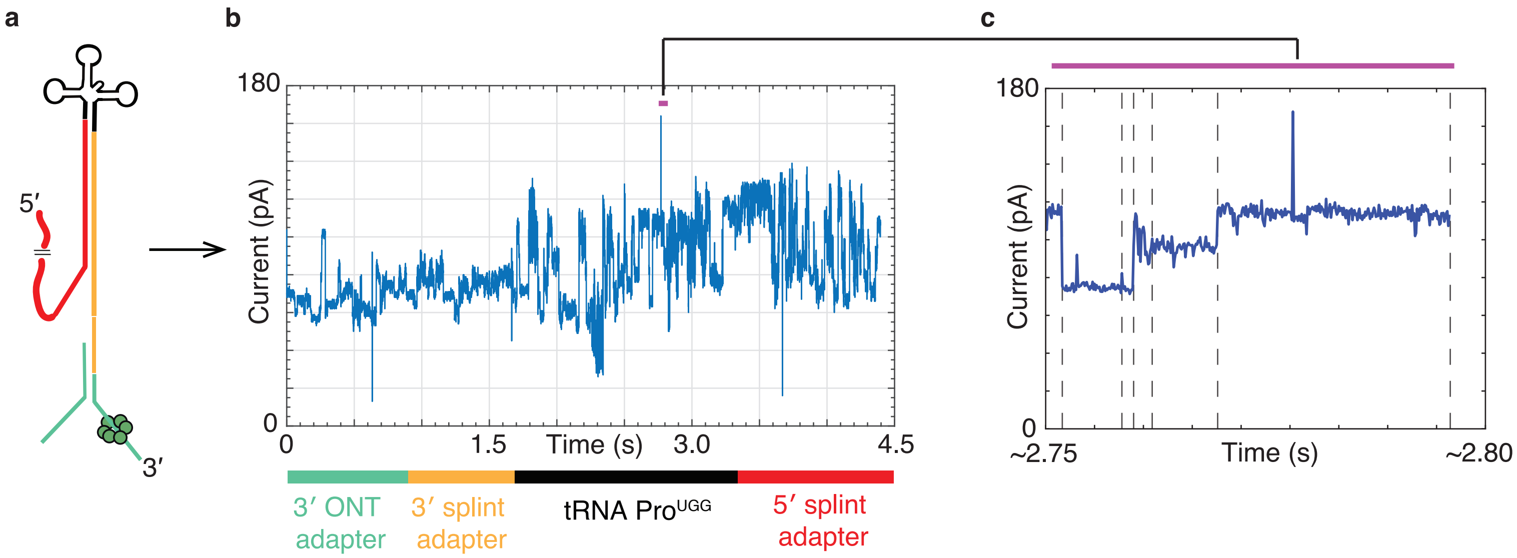

Supplementary Figure 8. Longer adapters enable ionic current analysis using Nanopolish. An illustration of the adaptation method, derived from Figure 1 but using a longer RNA-based splint adapter. The 5′ adapter is in red (120 nts), the 3′ adapter in yellow (46 nts), and the standard ONT adapters in green. b) Ionic current trace from a single tRNAPro(UGG) molecule with long adapters. c) Nanopolish generated segmentation of the ionic current from panel b. The region between each pair of vertical dashed lines represents an ionic current segment associated with a 5-mer, assigned by Nanopolish.

SUPPLEMENTARY REFERENCES

1. Giaever,G., Chu,A.M., Ni,L., Connelly,C., Riles,L., Véronneau,S., Dow,S., Lucau-Danila,A., Anderson,K., André,B., *et al.* (2002) Functional profiling of the Saccharomyces cerevisiae genome. *Nature*, **418**, 387–391.

2. Anderson,J., Phan,L., Cuesta,R., Carlson,B.A., Pak,M., Asano,K., Björk,G.R., Tamame,M. and Hinnebusch,A.G. (1998) The essential Gcd10p-Gcd14p nuclear complex is required for 1-methyladenosine modification and maturation of initiator methionyl-tRNA. *Genes Dev.*, **12**, 3650–3662.

3. Boccaletto,P., Stefaniak,F., Ray,A., Cappannini,A., Mukherjee,S., Purta,E., Kurkowska,M., Shirvanizadeh,N., Destefanis,E., Groza,P., *et al.* (2022) MODOMICS: a database of RNA modification pathways. 2021 update. *Nucleic Acids Res.*, **50**, D231–D235.

4. Jones,J.D., Simcox,K.M., Kennedy,R.T. and Koutmou,K.S. (2023) Direct sequencing of total tRNAs by LC-MS/MS. *RNA*, **29**, 1201–1214.

5. Behrens,A., Rodschinka,G. and Nedialkova,D.D. (2021) High-resolution quantitative profiling of tRNA abundance and modification status in eukaryotes by mim-tRNAseq. *Mol. Cell*, **81**, 1802–1815.e7.

6. Cozen,A.E., Quartley,E., Holmes,A.D., Hrabeta-Robinson,E., Phizicky,E.M. and Lowe,T.M. (2015) ARM-seq: AlkB-facilitated RNA methylation sequencing reveals a complex landscape of modified tRNA fragments. *Nat. Methods*, **12**, 879–884.

7. Cooley,L., Appel,B. and Söll,D. (1982) Post-transcriptional nucleotide addition is responsible for the formation of the 5’ terminus of histidine tRNA. *Proc. Natl. Acad. Sci. U. S. A.*, **79**, 6475–6479.
